# Cyclic Voltarefractometry of Single TiO_2_ Nanoparticles in Large Ensembles in Nonaqueous Electrolyte

**DOI:** 10.1021/acs.analchem.4c04181

**Published:** 2025-01-06

**Authors:** Veronika K. Laurinavichyute, Shavkat Nizamov, Vladimir M. Mirsky

**Affiliations:** Nanobiotechnology Department of the Institute of Biotechnology, Brandenburg University of Technology Cottbus-Senftenberg, Universitaetsplatz 1, Senftenberg 01968, Brandenburg, Germany

## Abstract

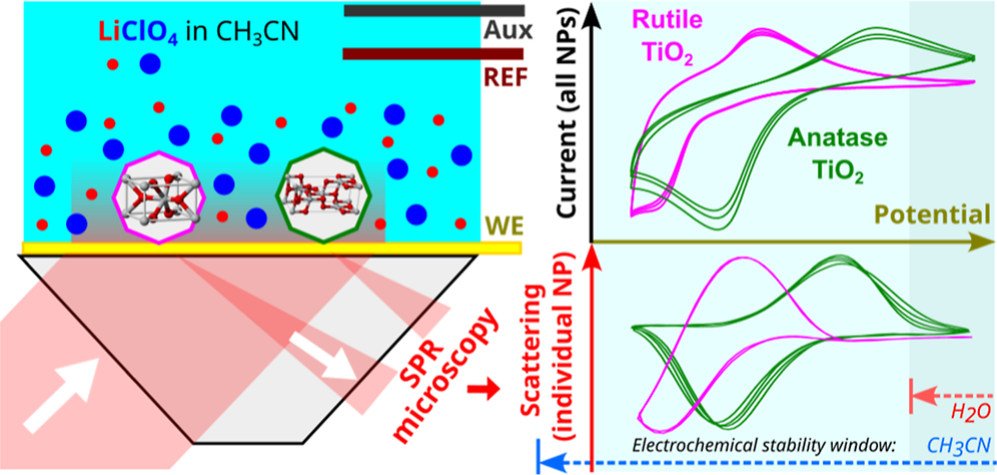

Single nanoparticle (NP) cyclic voltarefractometry (CVR), realized as wide-field surface plasmon resonance microscopy (SPRM) in combination with potential cycling, has been proposed and applied to the in situ study of TiO_2_ NPs. Electrochemical activity of TiO_2_ is mainly observed outside the electrochemical stability window of water. Therefore, the response of individual anatase (a-TiO_2_) and rutile (r-TiO_2_) NPs adsorbed on a gold layer was studied in 0.25 M LiClO_4_ acetonitrile solutions. The use of acetonitrile allows us to exploit a much wider potential window compared to water, while due to the almost identical refractive index (*n*_D_ = 1.344 and 1.333 for acetonitrile and water, respectively), the conditions of the SPR are not changed. This greatly expands the variety of electrochemical reactions that can be studied by SPR techniques. Cyclic polarization of a-TiO_2_ and r-TiO_2_ NPs results in pronounced electrochemical and optical responses around −1.55 V and around −1.8 V vs Fc^+^/Fc, respectively. This specific optoelectrochemical response allows them to be distinguished from other NPs. Based on this difference in characteristic potentials, a mixture of a-TiO_2_ and r-TiO_2_ NPs can be analyzed by CVR as well. The proposed correction algorithm compensates for the drift in the SPRM background caused by the accompanying formation of insoluble compounds and separates the optical response of the NPs out of the background. The results obtained in the study of this complex system demonstrate the capabilities of the developed analytical method. The CVR can be applied to the quantitative analysis of many other types of NPs in nonaqueous solutions, providing information on the electrochemical properties of each individual particle on the electrode surface.

Surface plasmon resonance microscopy (SPRM) is a type of optical microscopy under SPR conditions that allows an individual detection of adsorption/desorption of nanoparticles (NPs) and the rough quantification of their optical scattering.^1^ In general, optical microscopy and optical methods are rarely considered for the chemical identification of NPs unless they employ spectroscopic characterization such as Raman spectroscopy. However, spectroscopic approaches are difficult to combine with optical microscopy, and SPRM is no exception. A combination of SPR and surface enhanced Raman spectroscopy (SERS) microscopy is known but has not been widely adopted.^2,3^ Fortunately, SPRM can be seamlessly extended by both chemical and electrochemical methods. In this way, the state and response of adsorbed individual NPs can be monitored by SPRM under variations of chemical or electrochemical conditions. Thus, SPRM can also be used to study the chemical^4−6^ or electrochemical^7−10^ transformation of solid or gel NPs. The monitoring of nanoscale electrochemical processes by SPRM allows differentiation between adsorbed NPs with different redox behaviors, for example, metallic NPs such as Ag and Cu.^7−9^ However, the redox processes of many oxides or polymeric NPs take place outside the electrochemical stability window of water. Its width is thermodynamically defined as 1.229 V, but the actual potential range depends on the pH, electrolyte, type of electrode, etc. In practice, the stability window can be extended or moved somewhat. At neutral pH in dilute aqueous solutions, Pt electrodes, and Ag/AgCl reference electrode, it is approximately from −0.65 to +0.58 V. Therefore, it would be desirable to perform electrochemically assisted SPRM measurements in nonaqueous solvents with a much wider range of potentials available.

However, changing from one solvent to another is not as simple as it may seem. The majority of SPR instruments are designed and built for aqueous solutions because of their primary application in bio- and chemo-sensors. The refractive index (RI) of the solution defines the range of SPR conditions (i.e., wavelength and angle of incidence of the illuminating light) in which the SPR sensor can be used.

In order to maintain compatibility with existing SPR instrumentation, the choice of nonaqueous solvent is determined by its RI as another selection criterion. Given the extreme sensitivity of the SPR to the effective RI, its large change can easily push the SPR conditions beyond the optical design range. Therefore, the RI of the nonaqueous solvent should be as close as possible to that of water (*n*_D_ = 1.333). Thus, acetonitrile (ACN) (*n*_D_ = 1.344) seems to be the most natural choice. ACN is one of the most widely used and well-known solvents in electrochemistry.

In fact, there are only a few works where SPR has been performed in ACN, e.g., for the detection of testosterone,^11^ for the study of PEDOT electropolymerization^12^ or for the electrochemical study of hydroquinone–benzoquinone.^13^ Although the latter works illustrate the use of ACN in SPR in combination with electrochemistry, they were still done within the water stability potential window. No such examples could be found for processes at potentials outside the water stability window. As we will show further, despite the use of ACN as the solvent, the redox processes of water still remain one of the major challenges in the experimental study.

Our work focuses on oxide NPs, namely, titanium dioxide, which are of interest because of their extensive use in nanotechnology^14,15^ but the lack of analytical techniques for in situ identification. Besides many interesting features, the TiO_2_ NPs are also known for their electrochemical applications, including Li–ion batteries,^16^ solar cells,^17^ and electrocatalysis.^18^ There are several polymorphs of TiO_2_ (the most known being rutile, anatase, and brookite), each with specific physical and electrochemical properties. Therefore, the detection, quantification, and differentiation of TiO_2_ NPs is an important and challenging analytical task. In many real-world cases (e.g., environmental samples, trace concentrations, and complex matrices), this usually assumes the application of highly sophisticated, time-consuming, and expensive techniques, such as transmission electron microscopy, single particle inductively coupled plasma mass spectroscopy, etc.

The identification of TiO_2_ NPs by their electrode reaction is an alternative analytical approach. The electrode reaction of TiO_2_ at very negative potentials is accompanied by the reduction of H^+^ ions,^19^ which makes it difficult to study in aqueous solutions due to the overlap of electrochemical signals. On the other hand, the electrochemical behavior of TiO_2_ in nonaqueous solvents has been actively studied in recent years due to its wide application. It has been shown that the electrode reaction of TiO_2_ is closely related to the charge-compensating cations. Upon reduction, the charge compensation can be realized both by (i) accumulation of cations on the surface of the NP or (ii) intercalation of small cations (such as Li^+^) into the bulk of the particle
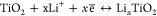


These two processes can proceed differently for different TiO_2_ polymorphs. The intercalation energy of charge-compensating cations is strongly influenced by the crystallographic structure of TiO_2_, that is why different TiO_2_ polymorphs exhibit electrochemical activity at different potentials.^20,21^ For example, lithium intercalation into rutile in propylene carbonate solutions occurs at the potential of ∼0.4 V more negative than the potential of intercalation into anatase.^22^ The modification B (bronze) is able to accommodate Li reversibly at potentials around 1.6 V in ACN, while for anatase this process occurs at ∼1.9 V vs Li/Li^+^.^23^ The amount of Li uptake can also vary, reaching 0.5 (Li_0.5_TiO_2_) for anatase^24^ and ranging from 0.15 to 0.85 for rutile, depending on the NP size.^25^

The idea of the current work is to use the electrochemical behavior of TiO_2_ NPs for the optical identification of individual NPs. To explore this possibility, we used wide-field SPRM (WF-SPRM) to follow the electrochemical transition of individual rutile and anatase TiO_2_ NPs in ACN during potential cycling. This novel method can be termed the cyclic voltarefractometry (CVR) of individual NPs. Furthermore, the reported difference in the electrochemical behavior of TiO_2_ NPs can be applied for the detection and speciation of the TiO_2_ polymorphs.

## Materials and Methods

Both synthesized and commercial samples of a-TiO_2_ (anatase) and r-TiO_2_ (rutile) NPs were used in the work. Details of the synthesis procedures, characteristics of the resulting particles, and preparation of NP suspensions are given in the Supporting Information#1.

All solutions were prepared using anhydrous ACN (VWR chemicals, max. 0.001 wt % H_2_O) and LiClO_4_ (battery grade, dry, 99.99%) and stored with 3 Å molecular sieves. Prior to measurements, solutions (without NPs) were filtrated successively through 200 μm Teflon and 20 nm Anotop syringe filters. Dry salts and glassware were stored under a vacuum with heating (∼60–80 °C) prior to measurements.

### Electrochemical Measurements

Electrochemical experiments were performed using the portable potentiostat/galvanostat PalmSens EmStat3 (PalmSens, The Netherlands). The three-electrode electrochemical cell for WF-SPRM measurements was mounted on the top of the prism with the sensor gold layer.

A planar glassy carbon electrode of 5 mm diameter was placed parallel to the prism surface at a distance of ∼3 mm and served as a counter electrode. The exposed geometric area of the gold electrode was limited by a rubber O-ring with an inner diameter of 4.3 mm. All potentials given below are vs Fc^+^/Fc. For more details, refer to Supporting Information#2.

### Cyclic Voltarefractometry

(WF-SPRM measurements under cyclic potential scanning). The principle of NP detection and analysis by WF-SPRM is described in detail elsewhere^1,26^ (see Supporting Information#3 as well).

SPR (and also WF-SPRM) is an ultrasensitive refractometry of the surface layer^1^ whose thickness is limited by the penetration depth of the evanescent wave (about 170 nm for 635 nm laser).^27^ In this work, WF-SPRM was performed simultaneously with cyclic voltammetry during cyclic potential scanning. Therefore, this approach can be called CVR. The plots of differential SPRM image intensity versus the applied potential can be referred to as voltarefractograms. This short and concise definition helps to distinguish them from purely electrochemical CV curves (voltammograms, shortened from voltamperometric curves), which have similar meanings and sometimes a similar shape. Voltarefractograms can be determined for the entire surface or for some defined spots on the surface (e.g., for individual NPs). For the determination of voltarefractograms from WF-SPRM records, the authors developed the interactive software for GPU accelerated data processing. A detailed description and analysis of the data processing pipeline will be published elsewhere. The main details and implementation are given in Supporting Information#3.

The numerical modeling of the SPR curves was carried out using the PyMoosh package, which implements a scattering matrix formalism for a multilayered structure.^28^

## Results and Discussion

### Electrode Pretreatment and Its Effect on WF-SPRM

**Figure 1:**
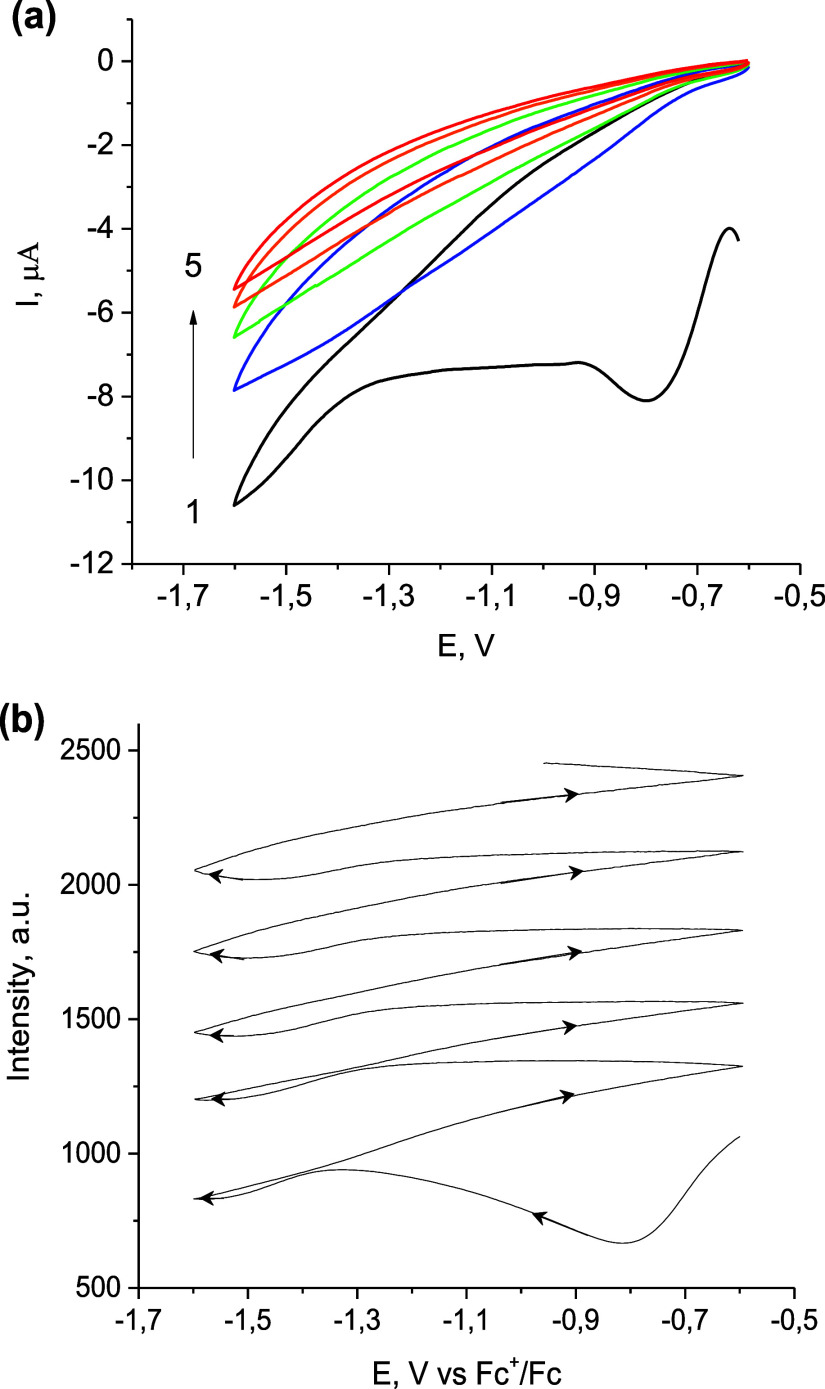
Cyclic voltammograms (a) and voltarefractograms of raw SPRM intensity (b) of Au electrode in 0.25 M LiClO_4_ acetonitrile solution at scan rate 20 mV/s.

After assembling the flow cell and filling it with electrolyte solution (0.25 M LiClO_4_ in ACN), the potential of the working electrode was cycled as in typical CV. After initial experiments, it became clear that the first cycles were of the utmost importance for the whole measurement. It was found that during the first cycle, a deposition of insoluble products (supposedly, lithium oxides or hydroxides, judging by the strong basic reaction of dried deposit in a water droplet on pH indicator paper) occurs on the gold electrode. In a typical starting procedure of the CV, the gold layer on the prism surface was cycled in the potential range of −0.6 to −1.6 V at a rate of 20 mV/s to stabilize the electrode surface. Furthermore, this should ensure the complete deposition of insoluble products so that this process does not interfere with subsequent CVs in the more negative range for the characterization of TiO_2_ NPs. The corresponding initial CV indicates the current peak at about −0.8 V at the first cycle, which usually decreased rapidly on subsequent cycles (Figure 1a). This process can be attributed to the reduction of the dissolved oxygen with a formation of superoxide ion (O_2_/O_2_^•–^) and further precipitation of lithium superoxide.^29^ The residual cathodic currents at more negative potentials can be ascribed to the reduction of the water traces with the formation of LiOH (insoluble in ACN)
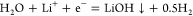


Due to rapid diffusion and high solubility of gases in ACN, their accumulation on the surface is not expected. The charge passed during the first cathodic scan was typically around 200–400 (up to 600) μC/cm^2^, corresponding to the formation of 1–2 monolayers of LiOH or Li_2_O_2_ (200 μC/cm^2^ per monolayer, assuming one electron transfer). Typically, the total cathodic charge flowed during this initial cycling corresponds to the formation of less than 2–5 nm of the passivating layer.

SPR is known and appreciated for its high sensitivity to the growth and adsorption of nanometer thick layers. The formation of such a thin layer on the sensor surface has a huge influence on the SPR conditions. This minor deposition results in a shift of the SPRM minimum position and a corresponding increase in the SPRM intensity at a fixed angle (Figure 1b). This process is most pronounced at the first cycle at potentials below −0.7 V (after O_2_ reduction) and is even less pronounced at the potentials of water reduction and during the subsequent positive potential sweep. Note that for the optimal signal-to-noise ratio, the SPR imaging conditions should be very close to the specular SPR minima.^30^ In this case, the light intensity from the sensor in SPR conditions is minimal. Accordingly, the camera’s exposure settings (image brightness) can be set as high as possible to achieve a dynamic range just about 3–4 times above the residual reflectivity. This dynamic range setting improves the signal-to-noise ratio and allows detection of weak SPR signal changes.^30^ However, if the changes in the SPR signal are too large, the selected dynamic range of the camera will be overflown. The overall change in the SPRM intensity during the first 5 cycles is so large that the change of imaging conditions becomes inevitable. Since the signal-to-noise ratio is determined by the ratio of the weaker signal to the stronger residual reflectivity, it is inevitable to adjust the measurement angle closer to the actual SPR minima. Accordingly, after a few initial stabilization voltage cycles, the angle of incidence was adjusted to close to the new SPR minima. This in turn requires an image position and focus adjustment followed by camera exposure stretching to cover the new dynamic range of image intensities.

Depending on the preparation of the solutions (e.g., residual water content in ACN), the formed layer can be much thicker than a few nanometers. As this shifts the SPR curve too much and decreases the signal-to-noise ratio anyway, no further SPRM measurements were taken in these cases. New solutions and gold-coated prisms were prepared, and the same procedure was repeated until the influence of the initial blocking layer on the SPRM was tolerable.

A small but constant increase in SPRM intensity was also observed during further cycling in a wider potential range (down to −2 V). The observed electrode passivation and corresponding SPRM minimum shift accompany the entire measurement, making it difficult, if not impossible, to accurately attribute the SPRM signal intensity changes to RI changes due to physical processes occurring.

Note that in a typical experiment, the electrode passivation significantly affects the optical signal but has almost no effect on the voltammogram of the probe molecule (ferrocene, see Figure S4, Supporting Information). Only prolonged cycling in a wide potential range leads to gradual blocking of the electrode surface, disappearing of the electrochemical signal of ferrocene, and shifting of the SPR minimum out of the experimentally accessible angular range.

In a typical experiment, the initial shift of the SPR curve during the pretreatment stage was around 1.3 ± 0.4° (with preadsorbed anatase NPs tending to have lower values than for rutile). Shifts above 2° were considered unacceptable, and the measurements were restarted with new samples (see above).

To estimate the influence of the insoluble deposits, SPR curves were simulated numerically. The overview of the available literature data (see Supporting Information#4) shows that the RI of gold varies greatly, depending mainly on the thickness of the gold and the method of preparation. Accordingly, absolute calculation of SPR curves and precise fitting of them to measured data do not seem to be reasonable. Nevertheless, the relative changes of the SPR curves should be quite independent of the value of RI of gold. There is also considerable variation in RI for Ti (interlayer). However, due to its small thickness and weaker metallic properties at this wavelength, it has little effect on the SPR curve (other than to reduce the overall reflectivity). Interestingly, at 640 nm, the Li as a metal has similar optical properties to gold itself. The chosen experimental conditions do not assume the deposition of pure Li on the gold surface, but it is worth considering.

**Figure 2:**
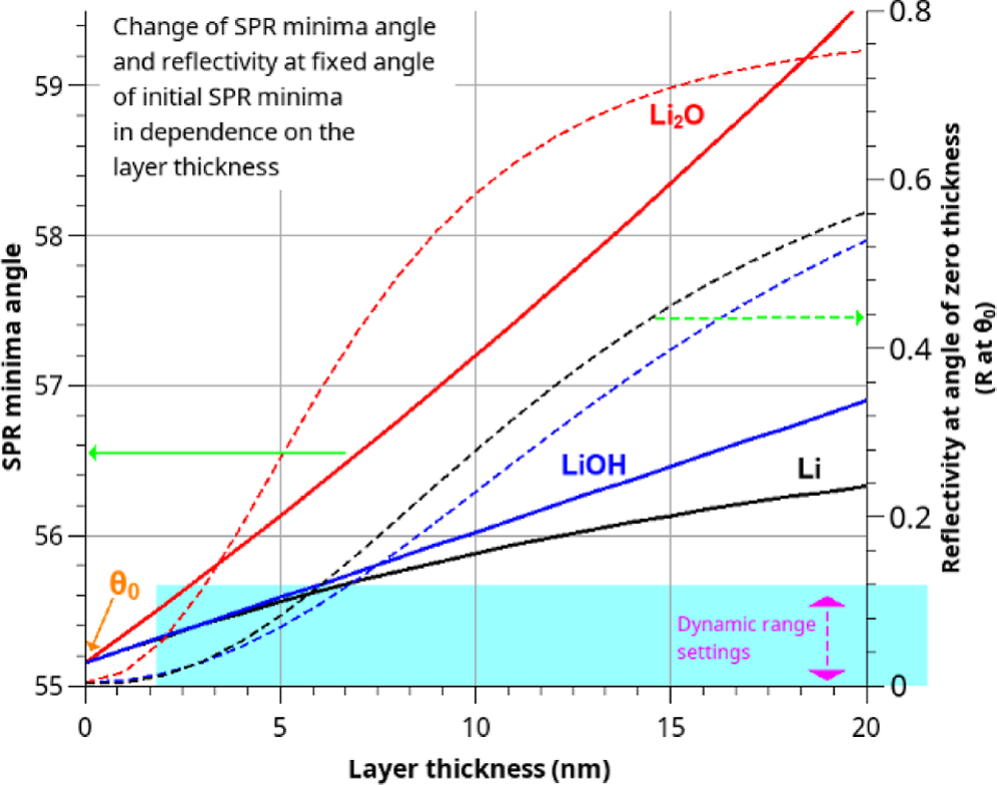
Change in SPR conditions (angle of minima in the left scale and change of reflectivity at the angle) as the Li/Li_2_O/LiOH layer grows on the surface. Simulation details are provided in Supporting Information.

The results of the simulation of the SPR curves are listed in Figure 2. The graph shows the variation of the SPR minimum angle as a function of the thickness of the adsorbed layer (left scale, solid line). As it can be seen, the SPR minima angle shifts for ∼0.8° for a 10 nm of LiOH layer, whereas the same thickness of a Li_2_O layer induces an SPR minima angle shift of ∼2.0°. No literature data could be found on the RI of Li superoxide Li_2_O_2_. Judging from its higher density than Li_2_O, its RI should be higher than that. Correspondingly, its effect on the SPR curve at the same thickness should be even more pronounced. If the incidence angle by SPRM was set to the SPR minima angle, the SPR reflectivity will correspondingly increase from its minimum value as the layer grows (right scale, dashed lines). When the angle of incidence is set to the initial SPR minima, the reflectivity at this point increases from ∼0 to 0.2 and 0.6, respectively. However, as explained above, keeping the dynamic range as low as possible is essential for detecting weak SPR signal changes. If the camera imaging setting covers only 0.1 of the reflectivity, the image intensity is already exceeded at thicknesses above 5 nm for LiOH and 3 nm for Li_2_O. In real experiments, the residual reflectivity at the SPR minimum is much higher than zero due to the surface roughness and inhomogeneity of the gold layer. Moreover, the illuminating beam has a finite collimation and polarization ratio. Overall, the selection of optimal conditions in the presence of a growing blocking layer is challenging.

A comparison of the observed typical shift of SPR minima (0.6–1°), electrochemical estimations of passive layer thickness (3–5 nm), and our simulation of the SPR minimum shift (Figure 2) suggests that lithium oxide or peroxide are the most likely components of the passive layer.

**Figure 3:**
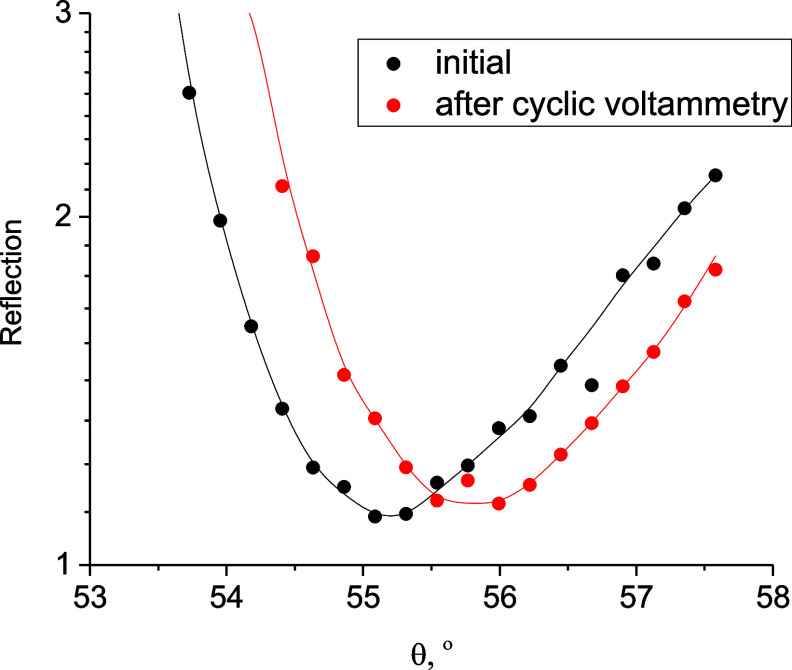
Angle dependencies of the reflected light obtained on the WF-SPRM setup before and after potential cycling in 0.25 M LiClO_4_ acetonitrile solution.

The observed constant shift in the SPR minima results in a pronounced drift of the background signal over time. As an example, the SPR angular curves before and after initial cycling are shown in Figure 3. Another important contribution to the background signal comes from the processes of charging and discharging of the electrical double layer,^31^ which also depends on the state of the electrode surface and the electrode potential. Both factors lead to a significant change in the optical background signal obtained at different measurement angles, to the appearance of loops and self-intersections (Supporting Information, Figure S7).

Since the interference from insoluble Li products is very high, the question is whether and how it can be minimized. Replacing lithium by a more soluble organic cation could diminish the surface passivation but would not allow the study of intercalation processes due to the cation size. The problem is that even anhydrous ACN contains a small amount of water. Literature data on ACN purification show that the lowest amount of water in ACN can be achieved by using molecular sieves. In this case, the remaining water in ACN reaches a level of 0.001%. In other words, the water content is 10^–5^ by the volume (or of the height of the electrochemical cell). Although this may seem to be a very low value, it means that each 1 mm thick layer of ACN contains 10 nm of water. This residual amount of water is sufficient to form a comparably thick layer of Li products. LiOH has a very low solubility in ACN. It is also difficult to remove dissolved O_2_ from ACN while maintaining the lowest possible water content. These impose strict conditions on the preparation and storage of all solutions used during measurements. As anhydrous ACN absorbs water from the air very quickly, contact with ambient air should be avoided as far as possible. Since a certain amount of water in solutions is unavoidable, its effect must also be taken into account in the data analysis.

### Signal Processing with Constantly Changing Background

**Figure 4:**
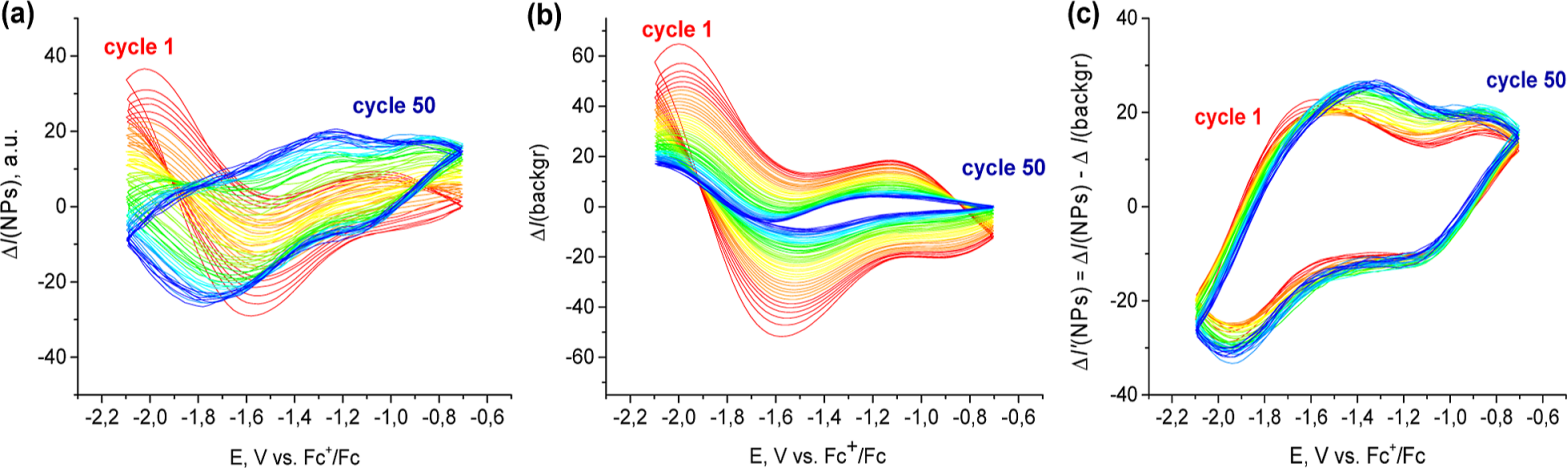
Mean (over *n* = 211 NPs) voltarefractograms measured during sequential cycling of the anatase + rutile mixture in 1 M LiClO_4_ in ACN at 200 mV/s. Local SPRM intensity change (a) was corrected for the global (background) changes (b), resulting in a corrected signal (c). Red, yellow, green, and blue colors correspond to the 1-st, 2-nd, 3-rd, and 4-th decade of the cycles, respectively.

To test the possibility of separating an optical signal from a constantly changing background, we performed long potential cycling (50 cycles at 200 mV/s) with adsorbed TiO_2_ NPs. The change in the optical signal in the center of the NP images (3 × 3 pixels) varies dramatically over the course of the cycling (Figure 4a). The initial increase in the signal at negative potentials becomes less pronounced with cycling and is followed by a decrease in the signal. The whole shape of the voltarefractograms changes completely.

At the same time, at the beginning of cycling, the background signal changed synchronously with the NP signal but with a larger amplitude (Figure 4b). With cycling, the intensity of the background signal decreases. Subtraction of the optical signal at the center of the images and the background signal intensity at each cycle gives a remarkably reproducible optical response (Figure 4c). The signal variation over 50 cycles does not exceed 15%. Thus, both irreversible and poorly controllable optical effects caused by ongoing surface passivation, i.e.

1) the drift of the SPR conditions and the resulting change in the voltarefractogram due to the deposit formation;
2) the change in the near-electrode layer during charging processes and the corresponding change in the voltarefractogram

can be taken into account by this background correction. Note that such dramatic changes were not observed in our short routine measurements (3–5 cycles per scan rate) and typical examples of background signal subtraction are shown in Figure S8.

### Ex Situ Electrochemical Characterization of TiO_2_ NPs

**Figure 5:**
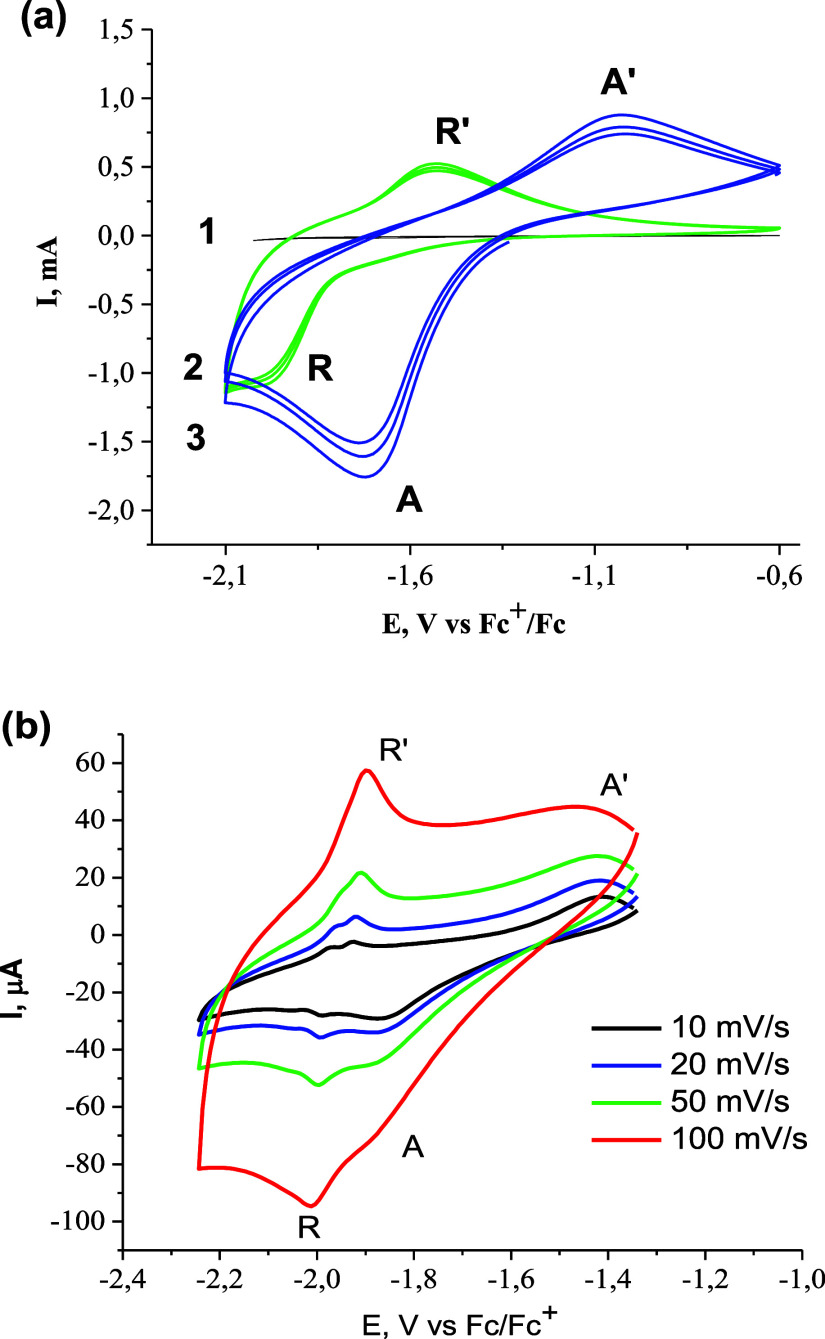
Cyclic voltammograms of individual types of NPs (a) and of a mixture of these various types of NPs (b) deposited on the gold electrode in 0.25 M LiClO_4_ acetonitrile solution. Curves 1, 2, and 3 in the panel (a) correspond to the uncoated electrode, to the electrode coated with r-TiO_2_ and to the electrode coated with a-TiO_2_ NPs. Scan rate of 10 (a) or 10–200 mV/s (b). Mass of TiO_2_: ∼25 (a) or 50 μg (b).

Cyclic voltammograms obtained on gold electrodes coated with either a-TiO_2_ or r-TiO_2_ NPs show broad peaks with large peak-to-peak separation at around −1.65 and −1.9 V, respectively (Figure 5a).

The voltammograms of the electrode, containing a commercial mixture of a-TiO_2_ and r-TiO_2_ NPs, exhibit two pairs of irreversible redox processes at approximately the same potentials: *A*/*A*′ at about −1.55 and *R*/*R*′ at −1.8 V (Figure 5b). Interestingly, the *R*/*R*′ redox process splits into two narrow overlapping peaks at low scan rates, similar to cyclic voltammograms reported in the literature for TiO_2_–B polymorph.^32^ This splitting and the overall higher reversibility of the redox processes observed for the commercial mixture can be attributed to their smaller particle size (see Supporting Information, Table 1).

### In Situ Characterization of TiO_2_ NPs by CVR

It was found that the reduction of TiO_2_ in ACN is coupled to the intercalation of Li^+^ cations or to reversible adsorption of charge-compensating cations^33^ (accompanied by anion expelling from the diffusion layer). The redistribution of ions near the sensor surface changes the effective RI within the penetration depth of the evanescent field. It is important to note that anions and cations do not contribute equally to the RI of compounds. This inequality of the partial RIs of anions and cations can be used, for example, to visualize surface charges by WF-SPRM.^34^

Unfortunately, the separate contributions of anions and cations can be rarely found in published works. The detailed consideration of this issue (see Supporting Information#5) shows that ClO_4_^–^ anions have the main influence on the effective RI. At the used concentration level, the Debye length (corresponding to the thickness of the ion depletion layer) is less than 1 nm and therefore negligible compared to the penetration depth of the surface plasmons. Consequently, the total amount of excess/expelled ions roughly corresponds to the surface charge.

For negative surface potentials, it can be assumed that all of the perchlorate anions are expelled from the approximately 1 nm thick layer. Concurrently, the concentration of lithium cations was significantly increased. However, due to its about 175 time smaller polarizability and molar refractivity in comparison to that of the perchlorate anion (see Supporting Information#5), these have a negligible impact on the effective RI of the depletion layer. Therefore, applying a negative potential to the SPR sensor surface reduces the RI within the 1 nm layer adjacent to the sensor. The maximum possible RI reduction in this depletion region can be estimated as down to the RI of the pure solvent.

The results of the numerical analysis of the SPR conditions indicate a decrease in the effective RI and a shift in the SPR minima to the left. However, the overall effect is surprisingly low. Even 5 nm of fully depleted (pure) ACN with the background of ACN + 250 mM LiClO_4_ shifts the SPR angle by only −0.015°. Notably, the adsorption of 0.7 nm LiOH has a similar effect but in the opposite direction.

This depletion mode assumes that the surface is fully accessible by solvent and ions. In the event of steric hindrance, for instance, in the form of an adsorbed layer of LiOH, the depletion region should expand in thickness accordingly, but not in total amount of ions or corresponding optical effect.

In turn, the intercalation of Li^+^ into TiO_2_ with the formation of Li*x*TiO_2_ results in a reduction of the RI by 0.65 (from 2.42 down to 1.77, at 650 nm).^35^ Therefore, both electrochemical processes are expected to decrease the local RI.

**Figure 6:**
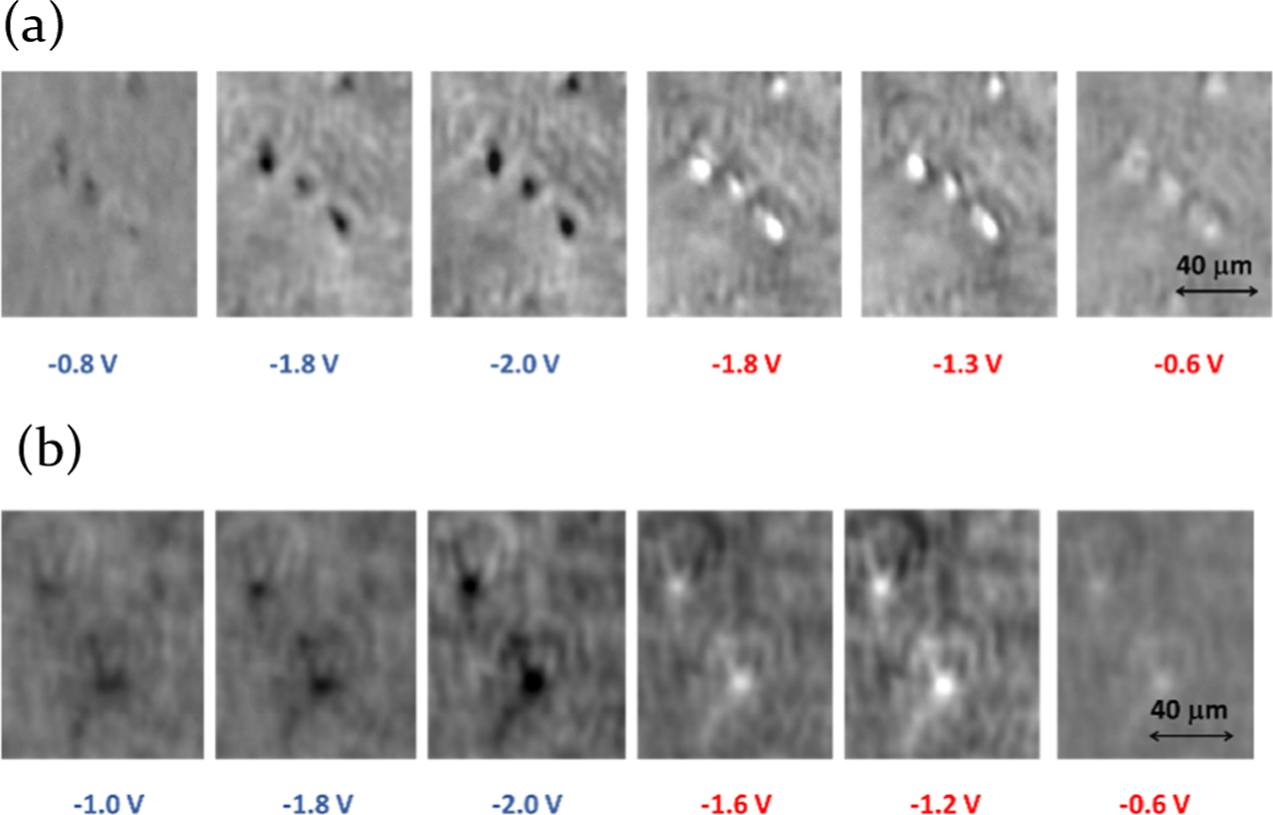
Typical SPRM images of a-TiO_2_ (a) or r-TiO_2_ NPs (b) in 0.25 M LiClO_4_ acetonitrile solution at different potentials. Potential values are represented by color, with negative scans displayed in blue and positive scans in red.

**Figure 7:**
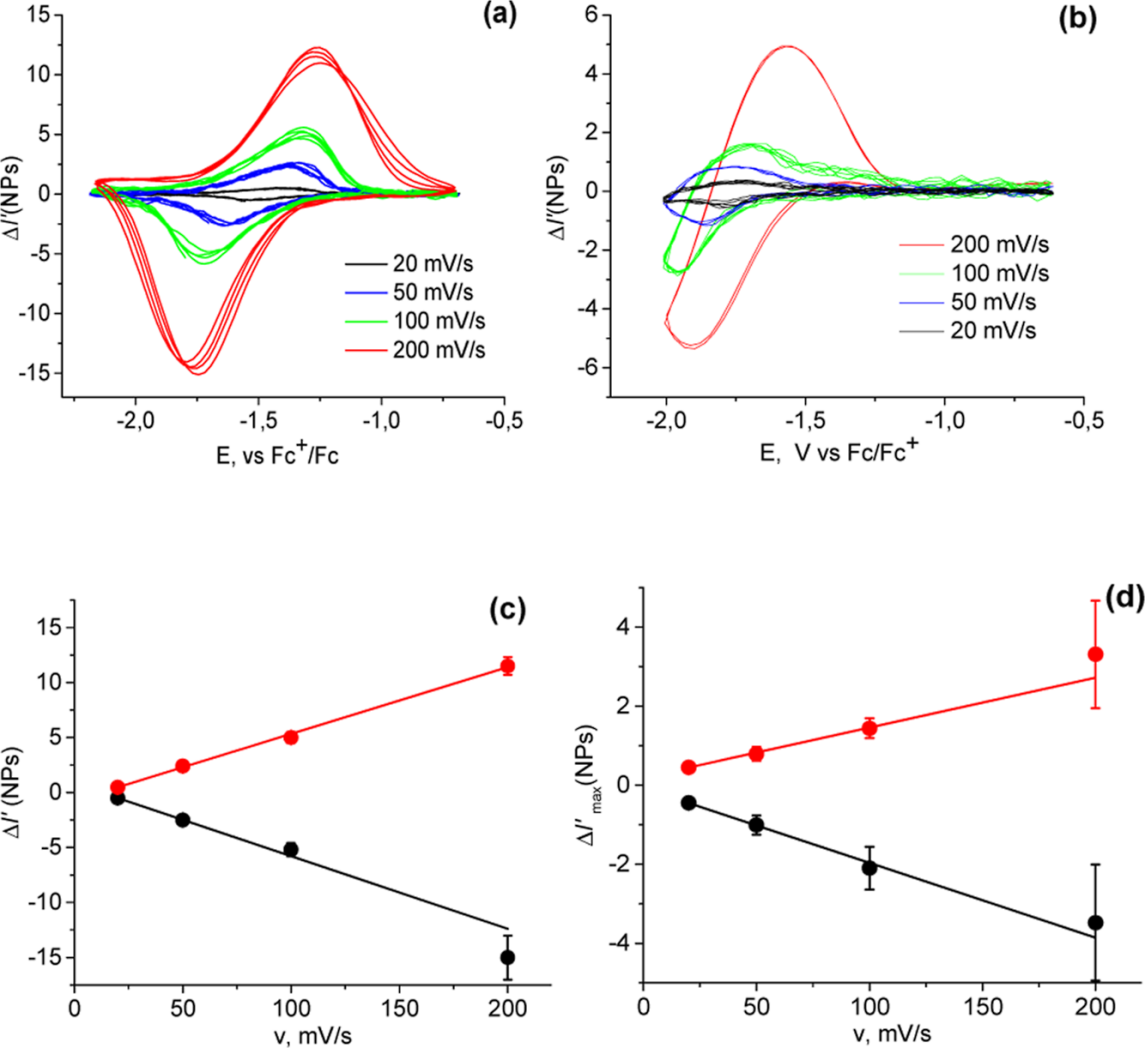
Mean (*n* = 198 (a) and 436 and (b) NPs) cyclic voltarefractograms (a,b) and corresponding dependences of their peak values on the scan rate (c,d), obtained for a-TiO_2_ (a,c), and r-TiO_2_ (b,d) NPs in 0.25 M LiClO_4_ acetonitrile solution at scan rates of 20–200 mV/s.

The averaged voltarefractograms obtained for individual adsorption sites indeed demonstrate the decrease in the local RI at sweeping the electrode potential in the negative direction and the increase in local RIs during the anodic potential sweep (Figures 6, 7), as previously hypothesized. These processes take place at around −1.55 V and around −1.8 V for anatase and rutile, respectively. These values are in good agreement with those obtained via *ex situ* electrochemical characterization. Note that the shape of the voltarefractogram in this case is less distorted by Ohmic losses, which are inevitable when testing a powder with poor intergranular conductivity (Figures 5 and S11).

As illustrated in Figure 7c,d, the corresponding peak intensities for single NPs increase linearly with the scan rate, thus indicating the capacitive nature of the observed processes. Additionally, it is noteworthy that intercalation of Li occurs as a result of ion diffusion within the solid crystal structure, a process that occurs at an essentially slower rate. Thus, the optical response is mainly due to the surface effects and is correspondingly weak.

Note that in the case of a low particle density on the gold surface (1–2 × 10^5^ NPs/cm^2^) and given experimental conditions, the electrochemical current of particles charging cannot be detected because of a greater contribution of the double layer recharging and side processes, such as water traces reduction (see Figure S9).

### In Situ Characterization of Adsorbed Rutile/Anatase Mixtures by CVR

**Figure 8:**
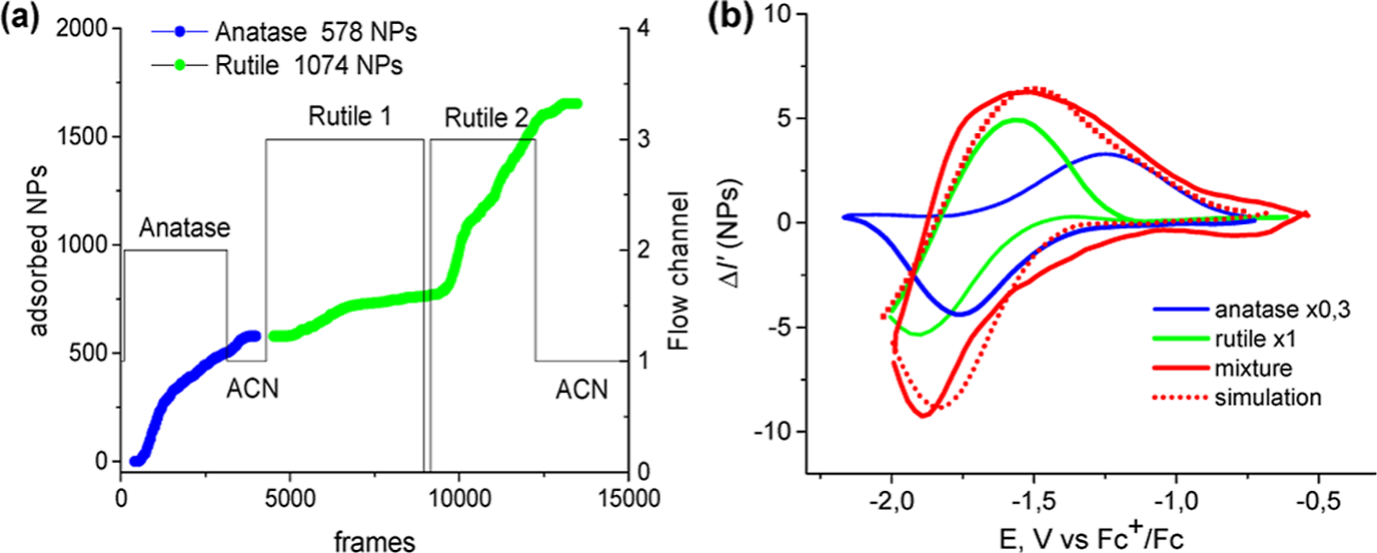
WF-SPRM in situ counting of adsorption of a-TiO_2_ and r-TiO_2_ NPs onto TMA-modified gold surface (a) and subsequent voltarefractograms obtained for these NPs in 0.25 M LiClO_4_ acetonitrile solution at scan rate 200 mV/s (b). Simulation (red dots) of experimental spectral signal (red line) was obtained by summation of individual spectral curves of a-TiO_2_ (blue) and r-TiO_2_ (red).

In order to regulate the surface concentration of the rutile and anatase NPs, the adsorption stage was conducted in situ through the successive pumping of a-TiO_2_- and r-TiO_2_ suspensions through the flow cell of the WF-SPRM device. The prior modification of the gold surface by *N*,*N*,*N*-trimethyl(11-mercaptoundecyl)ammonium chloride HS-C_11_NMe_3_Cl (TMA) results in the positive surface charge, which facilitates the adsorption of the negatively charged NPs.^36^ It is also noteworthy that the sensor surface in the used WF-SPRM is oriented horizontally, imaged from beneath, and exposed upside to the flow cell. Thus, the adsorption of NPs is also promoted by gravity. The number of adsorbing NPs was estimated visually from real-time differential SPRM images and controlled by the adsorption time. The adsorption of each NP was visualized for a few seconds as a bright spot at the corresponding location of the SPRM image of the sensor surface. Similarly, the desorption of NPs (if any) can be visualized as black spots at the same position. The desorption of NPs was in fact rarely observed; such desorbed NPs were excluded from further discussion. The flushing process with NP suspensions of a-TiO_2_ or r-TiO_2_ was continued until the desired number of NPs was adsorbed (Figures 8a and S10a).

Following the adsorption stage, the solution was replaced with a 0.25 M LiClO_4_ ACN solution, and electrochemical surface pretreatment was conducted (see above). The electrochemical pretreatment results in a significant alteration of the SPR conditions, necessitating the adjustment of the SPR incidence angle and SPRM focus. Then the potential was cycled in a wide potential range at different scan rate.

The application of slow scan rates (1–20 mV/s) resulted in a low amplitude of the optical signal for the TiO_2_ NPs. Consequently, only the data obtained at higher scan rates (50–200 mV/s) were subjected to analysis. It is important to note that the rate limit in question is determined by the specific process or object under study, in this case, TiO_2_. For other systems, such as LiFePO_4_ in ACN, at scan rates as low as 0.1 mV/s, a distinctive optical response was observed (to be published elsewhere). Although it was not possible to identify individual TiO_2_ NPs with different voltarefractometric responses, the mean voltarefractometric response demonstrates a complex shape, which roughly corresponds to the sum of individual optical signals of anatase and rutile (Figures 8b and S10b). An improvement of the signal-to-noise ratio for separate NP voltarefractograms is needed for their individual discrimination.

**Figure 9:**
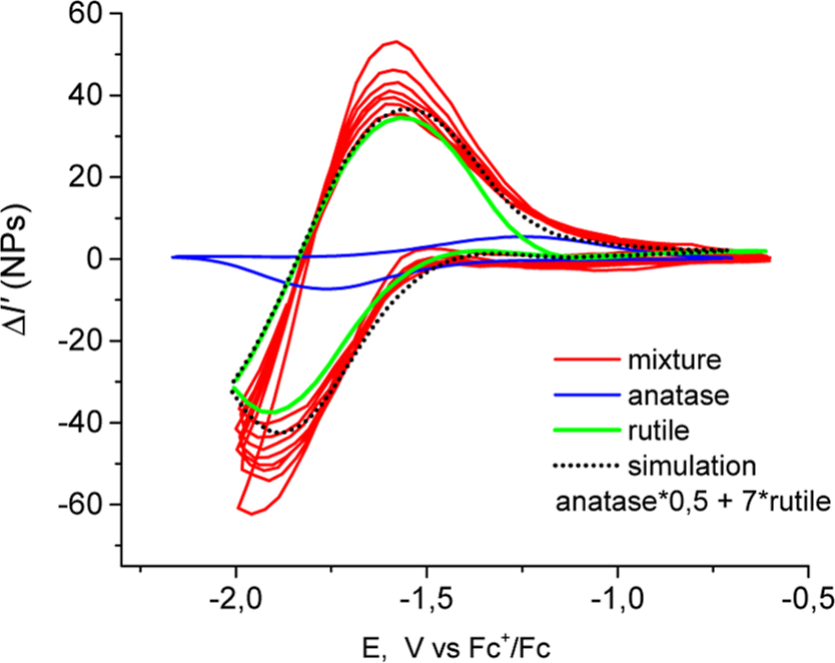
Voltarefractograms of the 1:1 rutile to anatase ratio TiO_2_ NP samples (commercial mixture enriched by rutile) (red curve) and its simulation (red dots) obtained by summation of individual voltarefractograms of a-TiO_2_ (blue) and r-TiO_2_ (green).

The voltarefractometric response of a sample of a commercial mixture of TiO_2_ NPs enriched by rutile NPs, with a resulting anatase/rutile particles number ratio of approximately 1:1, has a simpler form than other mixtures (compare the red curves in Figures 8 and 9). Its separation into a weighted sum of rutile and anatase contributions shows that the main optical signal is due to rutile (green curve, Figure 9). Only a small fraction of the signal can be attributed to anatase (blue curve, Figure 9). Figures 8 and 9 demonstrate that the mean voltarefractogram obeys the additivity of individual NP types. In the same time, the WF-SPRM signal depends almost linearly on the particle size.^7,37,38^ This size dependence is less dramatic than with DLS or NTA, but still leads to some masking effect. Correspondingly, the voltarefractometry of 30 nm anatase particles can be essentially diminished in the presence of two times bigger rutile particles. The difference in the surface area and properties of anatase and rutile NPs may also lead to a difference in absolute values of surface charging effects.

## Conclusions

In this work, CVR was suggested to monitor electrode reactions occurring on individual anatase and rutile NPs. These particular types of TiO_2_ NPs were adsorbed on the surface of a gold sensor layer. Their electrochemical conversions were observed using WF-SPRM. This is made possible by using ACN as a solvent. In comparison to water, it has one important feature: a much broader electrochemical potential stability range, while the value of RI is almost the same. This approach can potentially be extended to other solvents with a wide window of electrochemical stability. Since NPs are usually irreversibly adsorbed,^7,36^ the solvent replacement approach can be suggested. For example, NPs can be immobilized from aqueous suspensions and subsequently analyzed in ACN. In the current work, we really tried this approach successfully in initial experiments but had to exclude it later because of the distorting influence of trace amounts of water. Nevertheless, this methodology can be employed, particularly when the electrolysis of water is not a critical factor in the experimental system or when the cell thickness is sufficiently small that the total residual water is negligible.

It has been shown that cycling in a wide range of potentials in LiClO_4_ solutions in ACN leads to the irreversible passivation of the sensor. This phenomenon is attributed to the formation and deposition of various Li/H_2_O/O_2_ products that are insoluble in ACN. Consequently, a significant initial and later weaker but constant shift of the SPR curve occurs during such measurements.

The developed algorithm of optical background correction under the conditions of passive layer formation permitted the compensation of severe signal distortion caused by the transition through the minimum of the SPR curve. It makes the CVR in WF-SPRM conditions a promising approach to studying processes in nonaqueous electrolytes that are complicated by the formation of a new thin solid–electrolyte interface (SEI). The latter are typically formed in Li–ion batteries operating in nonaqueous electrolytes and composed of Li_2_O, LiF, Li carbonates, and organic compounds.^39^ The formation of the SEI with a nonpermanent composition complicates the study of Li intercalation/deintercalation processes.

The in situ cyclic polarization of a-TiO_2_ and r-TiO_2_ NPs adsorbed on a gold-coated prism results in pronounced optical responses in the adsorption spots around −1.55 V and around −1.8 V vs Fc^+^/Fc, respectively. This corresponds to the redox potential obtained by electrochemical measurements (Figures 5 and S10). The linear dependence of peak current/image intensity on scan rate indicates that predominantly surface charging/discharging processes occur.

The specific optoelectrochemical response of a-TiO_2_ and r-TiO_2_ allows them to be distinguished from the other NPs. Both self-synthesized and commercially available TiO_2_ NPs were used. In light of the observed discrepancy in the characteristic potentials associated with surface charging and discharging, an effort has been made to analyze a mixture of a-TiO_2_ and r-TiO_2_ NPs using WF-SPRM. Voltarefractometric response of mixtures can be separated into a weighted sum of their mean responses. For a-TiO_2_ and r-TiO_2_ mixtures of similar size NPs, a semiquantitative analysis of the mixture was possible.

These results demonstrate the synergy of optical and electrochemical analytical techniques and open the way for application of voltarefractometry for the analysis of individual NPs by their electrochemical redox reactions across a broad potential range.

## Supporting Information Available

Synthesis and characterization of TiO_2_ NPs and their suspensions; electrochemical measurements; wide-field SPRM; literature data on dielectric permittivities of Au, Ti, and Li; influence of ion redistribution on the RI near the sensor surface; correction of cyclic voltarefractograms of adsorbed TiO_2_ NPs for background; in situ CV of prism coated by a-TiO_2_ and r-TiO_2_ NPs; and ex situ CV of a-TiO_2_ and r-TiO_2_-modified gold electrodes: effect of scan rate (PDF)

## Abbreviations

a-TiO_2_: anatase
r-TiO_2_: rutile
NPs: nanoparticles
SPR: surface plasmon resonance
SERS: surface enhanced Raman spectroscopy
WF-SPRM: wide-field surface plasmon resonance microscopy
DLS: dynamic light scattering
NTA: nanoparticles tracking analysis
TEM: transmission electron microscopy
ICP-MS: inductively coupled plasma mass spectroscopy
RI: refractive index
CVR: cyclic voltarefractometry
CV: cyclic voltammetry
ACN: acetonitrile
TEAHSO_4_: triethylammonium hydrogen sulfate
TMA: *N*,*N*,*N*-trimethyl(11-mercaptoundecyl)ammonium chloride HS-C_11_NMe_3_Cl
