# Supplementary material for: Cyclic Voltarefractometry of Single TiO2 Nanoparticles in Large Ensembles in Nonaqueous Electrolyte
Source: Anal Chem. 2025 Jan 6;97(2):1160–9. doi: 10.1021/acs.analchem.4c04181 (PMC11755399; doi:10.1021/acs.analchem.4c04181)
Supplement: Supplementary file 1 — ac4c04181_si_001.pdf [file ac4c04181_si_001.pdf]

Supporting Information

Cyclic voltarefractometry of single TiO_2 nanoparticles in large ensembles in non-aqueous electrolyte

Veronika K. Laurinavichyute[1], ‡ Shavkat Nizamov[1*]‡, Vladimir M. Mirsky[1]

Brandenburg University of Technology Cottbus—Senftenberg, Nanobiotechnology Department of the Institute of Biotechnology, 01968 Senftenberg, Universitaetsplatz 1, Brandenburg, Germany

Table of Contents

1. Synthesis and characterization of TiO_2 nanoparticles and their suspensions 2

2. Electrochemical measurements 4

3. Wide-field surface plasmon resonance microscopy 5

4. Literature data on dielectric permittivities of Au, Ti and Li 7

5. Influence of ion redistribution on the refractive index near the sensor surface 9

6. Correction of cyclic voltarefractograms of adsorbed TiO_2 nanoparticles for background......................10

7. In situ cyclic voltammetry of prism coated by a-TiO_2 and r-TiO_2 NPs..11

8. Ex situ cyclic voltammetry of a-TiO_2 and r-TiO_2 modified gold electrodes: effect of scan rate 11

1. Synthesis and characterization of TiO_2 nanoparticles and their suspensions

Both self-synthesized and commercially available TiO_2 NPs were used in the current work.

The hydrothermal synthesis of **anatase (a-TiO_2)** was carried out according to the modified procedure [i]. Briefly, 1.7 ml of HF and 12 ml of H_2O_2 were added to 10.0 ml of $Ti(OBu)_4$ with stirring, and 8.18 g of the resulting orange suspension was transferred to a Teflon-lined autoclave (50 ml) and was heated at 180°C for 24 hours. The resulting product was washed with water and ethanol and then dried at 60°C for 24 h. 0.5473 g of the product was obtained, this corresponds to the yield of 67.4%. The phase composition was confirmed by XRD (Fig. S1a). The average particle size was 67 ± 23 nm according to TEM data (Fig. S2a), and 72 ± 25 nm in ACN suspension according to NTA.

Rutile (r-TiO_2) was synthesized according to the procedure [ii]. Briefly, 6.8 ml $Ti(OBu)_4$ was slowly added to 10.2 ml of HCl solution while stirring. Then, 23.8 ml of deionized water was added dropwise to the resulting clear sol, and the resulting mixture was stirred for three days. 20 ml of this mixture was then transferred to a Teflon-lined autoclave (50 ml) and heated at 220°C for 10 hours. The obtained compound was washed several times with water and ethanol and dried in air. The amount of r-TiO_2, obtained in this way was 0.54 g, corresponding to a yield of 67.91%. The single-phase nature of the sample was confirmed by X-ray diffraction (Fig. S1b). TEM shows a rod-like morphology of the synthesized r-TiO_2 crystallites with average length of 147 ± 66 nm and average width of 43 ± 12 nm (Fig. S2b,c). The mean hydrodynamic particle size in the dispersion in ACN was 68 ± 37 nm (NTA).

Individual a-TiO_2 (anatase) or r-TiO_2 (rutile) dispersions in acetonitrile were prepared by sonication of 1.5 mg of a-TiO_2 or r-TiO_2 in 2 ml of ACN containing 0.002 M triethylammonium hydrogen sulfate ($TEAHSO_4$), and subsequent 6000 times dilution of the resulting solution with the 0.002 M $TEAHSO_4$ solution in ACN.

The mixture of a-TiO_2 and r-TiO_2 was prepared from individual samples (A1, R1 or R2) or by dilution of commercial mixture (M). In the latter case the dispersion of 2 mg of a commercial mixture of rutile and anatase (Sigma Aldrich, < 150 nm, 33-37 wt.% in water) was dispersed in 4 ml of ACN containing 0.002 M $TEAHSO_4$. TEM shows that the dispersion contains nanoparticles starting from a few and up to 40 nm (Figure S2d). The phase composition of M was determined by XRD analysis to be 13 wt.% rutile and 87 wt.% anatase (Figure S1c).

The main characteristics of both own synthetic and commercial TiO_2 nanoparticles and their stock suspensions as determined by XRD, TEM and NTA is presented in Table 1.

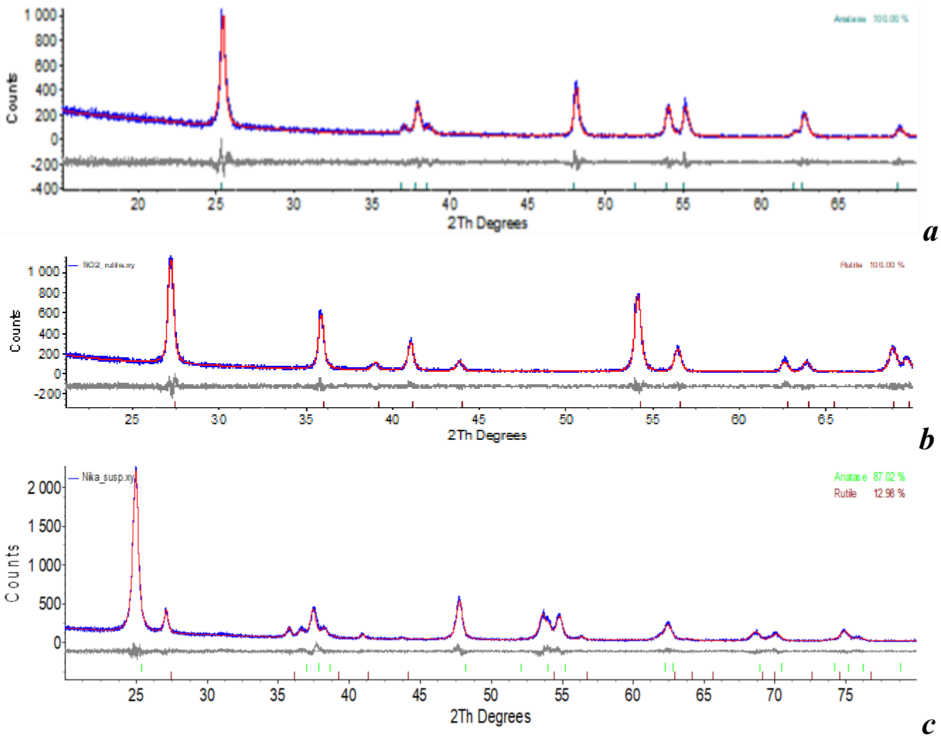

Fig. S1. XRD analysis of a-TiO_2 (a), r -TiO_2 (b) and a commercial mixture of a-TiO_2 and r-TiO_2 (c).

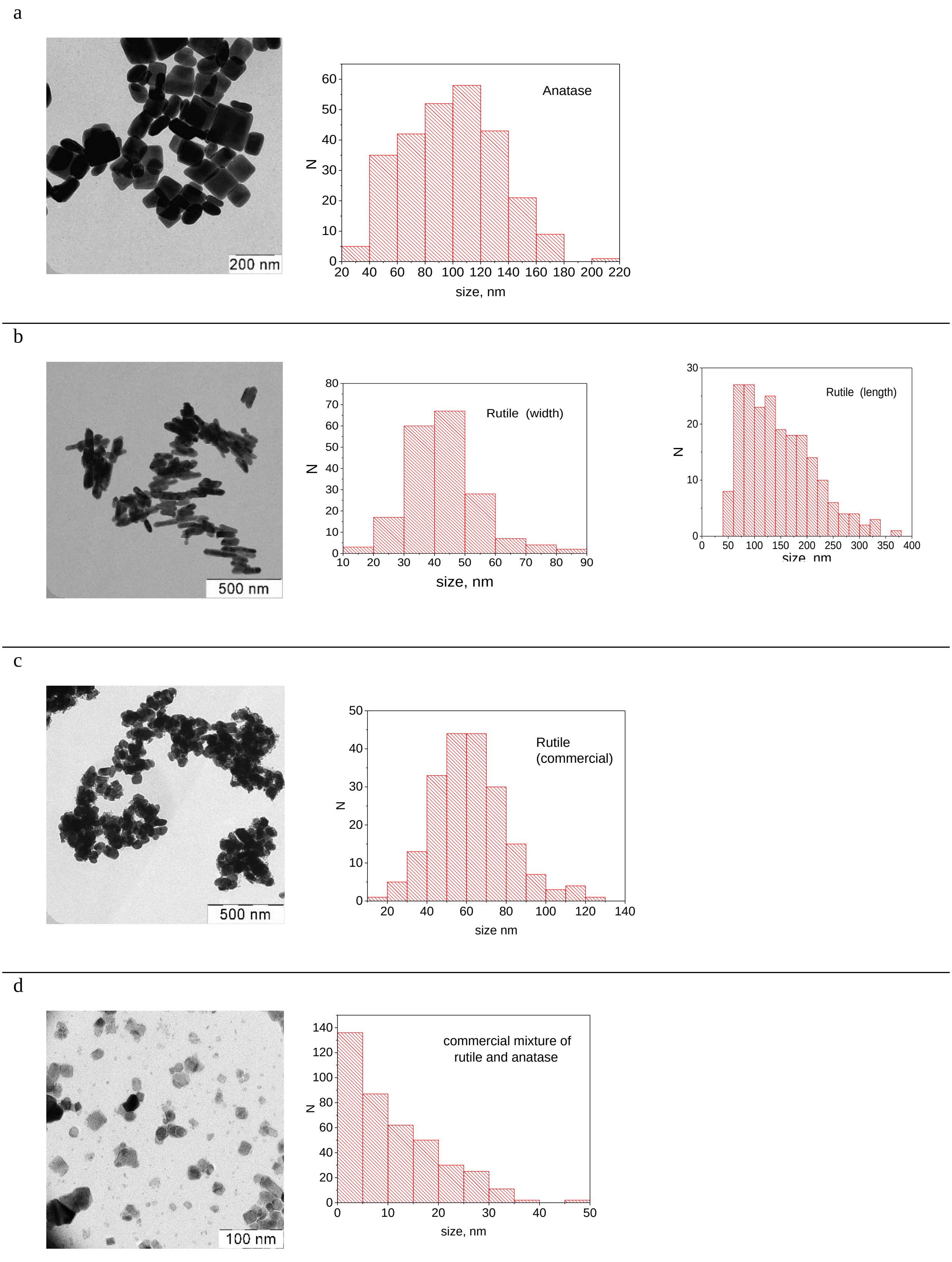

Fig. S2. Transmission electron microscopy images and size distributions of a-TiO_2 (a), r -TiO_2 (b), r -TiO_2 (commercial sample, c) and commercial mixture of rutile and anatase (Sigma-Aldrich, d).

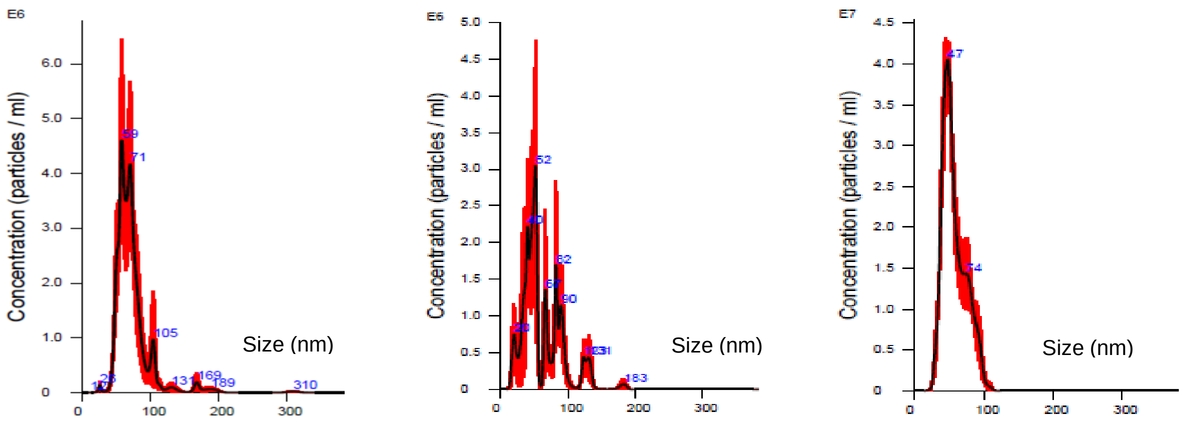

a B C

Fig. S3. Mean hydrodynamic size distributions of TiO_2 NPs measured by NTA in diluted acetonitrile stock suspensions of a-TiO_2 (a), r-TiO_2 (b) and commercial mixture of rutile and anatase (Sigma-Aldrich, c).

Table 1. Characterization of TiO_2 samples (for details see SI, sections 1 and 2).

Sample name	Phase composition (XRD)	Average size, nm			Concentration of stock suspensions, *10^8 NPs/ml
		XRD	TEM	NTA	NTA
A1 (anatase)	100 wt.% a-TiO_2	83 ± 14	99 ± 34	72 ± 25	1.40 ± 0.08
R1 (rutile)	100 wt.% r-TiO_2	44 ± 61	147 ± 66[&]<br>43 ± 12[#]	68 ± 37	1.0 ± 0.2
R2* (rutile)	r-TiO_2	-	62 ± 19	87 ± 43	0.87± 0.07
M** (mixture)	87 wt.% a-TiO_2<br>+ 13 wt.% r-TiO_2	30 ±40<br>47 ± 49	2 – 40	57 ± 17	13 ± 6

* Nanomaterials, ** Sigma Aldrich, & length, # width

2. Electrochemical measurements

Electrochemical experiments were performed using the portable potentiostat/galvanostat PalmSens EmStat3 (PalmSens, the Netherlands).The 3-electrode electrochemical cell for WF-SPRM measurements was mounted on the top of the prism with the sensor gold layer.

The gold-coated sensor prisms were preliminarily cleaned with freshly prepared "piranha solution" (1:3 v/v mixture of 30 wt. % H_2O_2/H_2SO_4 conc. *Caution: this solution reacts violently with most organic materials and must be handled with extreme care!*), thoroughly rinsed with water and ethanol, and dried at room temperature.

Gold wire or gold leaf was placed in contact with the gold layer on the prism and acted as a connector to working electrode.A planar glassy carbon electrode of 5 mm diameter was placed parallel to the prism surface at a distance of ~3 mm and served as a counter electrode. The exposed geometric area of the gold electrode was limited by a rubber O-ring with an inner diameter of 4,3 mm. All potentials given are vs. Fc^+/Fc.

Bare Au wire or Au wire coated with partially oxidized polypyrrole film was placed in the cell outlet and acted as a quasi-reference electrode. [iii] The polymer film was fabricated by cyclic voltammetry with the Au wire electrode in an acetonitrile solution of 10 mM pyrrole containing 0.25 M $LiClO_4$. The stability of the quasi-reference electrode was checked daily *in situ* by cyclic voltammetry in acetonitrile solution of 0.5 mM ferrocene, containing 0.25 M $LiClO_4$.

In order to calibrate the pPy/Au quasi-reference electrode and to check the degree of surface blocking by electrodeposited by-products, the cyclic voltammograms in acetonitrile solution with the addition of 0,5 mM ferrocene were measured at a scan rate of 50 mV/s after each series of experiments (Fig. S4).

For the *ex situ* electrochemical characterization of TiO_2 materials, a small amounts of stock solutions (corresponding to 20-50 µg of TiO_2) was placed on the gold wire surface and dried in a vacuum.

Adsorption of NPs onto the gold layer was performed *in situ* by pumping the diluted TiO_2 dispersion (1/1500) through the cell or *ex situ* by dropping 10-20 µl of the diluted stock dispersion of NPs (1/6000) onto the gold layer, washed with ACN and dried in vacuum.

All solutions and suspensions were pumped using a solenoid operated micropump (Biochem Valve, 130SP1220-1TP, 12 V-DC) at a flow rate of 0.5–1 mL/min.

All vessels used must be free of adsorbed water. This can be achieved by drying them in a vacuum oven. The fluidic subsystem of the WF-SPRM can be dried by pumping a large volume of dried ACN (10-20 ml) at a slow flow rate (0.1-0.2 ml/min). Such measures help to reduce the impact of residual water in the ACN, but a certain amount is unavoidable. Accordingly, these effects must also be taken into account in the data analysis.

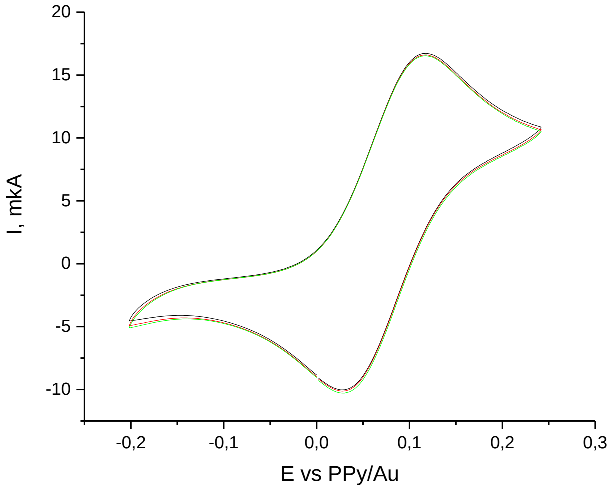

Fig. S4. Typical electrochemical response of the probe molecule (ferrocene) after spectroelectrochemical measurements. In situ cyclic voltammograms measured with a gold layer on the prism and adsorbed TiO_2 NPs in 0.25 M $LiClO_4$ acetonitrile solution containing 0,5 mM ferrocene. Scan rate: 50 mV/s.

3. Wide-field surface plasmon resonance microscopy

A 635 nm PM fibre-coupled laser diode (Wave Spectrum WSLP-635-040m-PM-PD) with current and temperature controllers (LDC205C and TED200C respectively, www.thorlabs.com) was used as the light source. The light was collimated into a parallel beam using a 16 mm focal length objective (MVL16, Thorlabs, www.thorlabs.com). The polarization ratio of the light beam was further improved using a 14 mm free aperture Glan polarizer (EksmaOptics, www.eksmaoptics.com). The polarization plane was set to *p*-polarization relative to the surface of a gold-coated SF-10 prism (n_D = 1.72). The thickness of the gold layer was 43-45 nm with a 3–5 nm thick titanium adhesive layer (www.phasis.ch). The light reflected from the gold surface was focused on the SONY XCL-SG510 camera by a proprietary optical system (EksmaOptics, www.eksmaoptics.com) with an input aperture of 18 mm and a focal length of 23 mm (NA = 0.39). According to the optical system designer, the optical resolution of the setup is ~2 µm, limited by diffraction. The camera incorporates a 2/3" Global Shutter CMOS Sony IMX250 monochrome image sensor. The image sensor has 2448 × 2048 square 3.45 µm pixels. One pixel corresponds to ~570 nm × 570 nm of visible surface (calibrated by the prism with an engraved 500 µm grid). The imaged area is ~1.4 × 1.2 mm (~1.7 mm^2, depending on the angle of incidence and image focus settings). The camera is connected to the MicroEnable 5 marathon ACL Framegrabber (Silicon Software) via a CameraLink interface.

The image processing pipeline is shown schematically in Figure S5. Images captured by Sony's XCL-SG510 camera were summed in short sequences, typically 4/8/12 or 16 frames long (depending inversely on the CV rate). This is done to reduce the storage bandwidth and capacity required to store images. The Sony XCL-SG510 camera produces a large stream of data: it was typically set at a rate of 64 frames per second, with each frame being 5 megapixels at 8-bit depth. This is equivalent to ~320 Mbyte/sec with these settings. Summing up a few consecutive frames and saving them as 16-bit TIFF images reduces manifold the storage requirements. TIFF images also embed some experimental details at the time of capture as metadata.

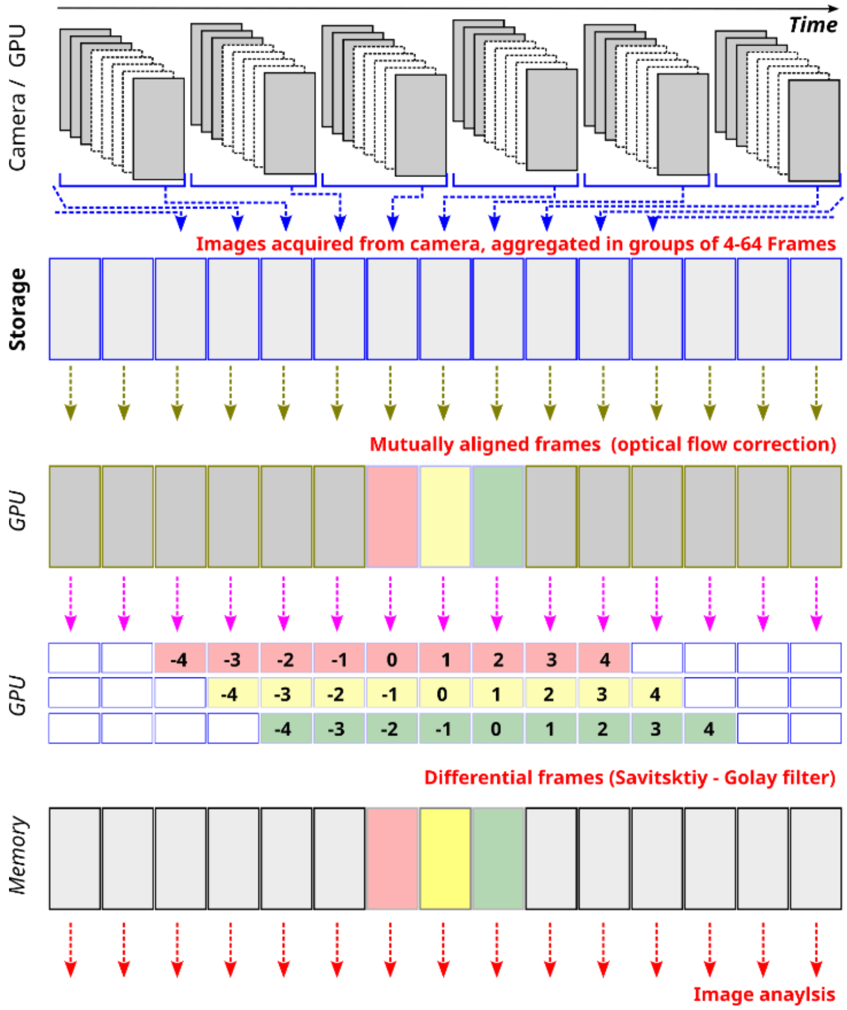

Fig. S5. Image processing pipeline for obtaining differential SPRM images.

TIFF image sequences were saved and used for analysis without further modification. The most informative way to proceed with the SPRM images is to analyze the image changes in response to some external perturbation (or simply over time). Savitsky-Golay (SG) filter was applied to quickly and effectively determine image changes. The SG filter can be used to smooth the original data using a polynomial of specified order or to calculate its derivatives. Since the SG is essentially just multiplying frames by some predetermined coefficient and summing them up, it can be parallelized very effective, especially with GPU accelerators. Depending on the time resolution of the experimental recording (effective frame rate after aggregation) and the expected response dynamics (scan rate of the CV), the length of the SG filter can be chosen accordingly. Obviously, the longer SG filters have a better smoothing but worse time resolution, and vice versa.

The SPRM setup has a number of optical elements that are either moveable/adjustable, or are aligned by spring loaded mounts. In addition, there are some fluctuation of air flows within the setup, external mechanical disturbances, laser light mode and intensity fluctuations etc. All of these cause slight transient distortions in the images obtained (optical drifts), which make it difficult to analyze the changes in the image. That’s why the optical drifts in the SPRM images are corrected before the SG filter is applied. SPRM images are aligned to sub-pixel resolution using optical flow

correction. This is done using the direct neighborhood of frame under consideration. The same range of frames is taken as for the SG filter, and frames within this range are mutually aligned to subpixel resolution. This reduces the noise due to transient optical drift in the differential frames obtained by applying the SG filter.

All data processing was done on the fly in computer memory using an Nvidia GPU accelerator. The generated differential images were provided for further image analysis. To facilitate comparison of different recordings, the intensities of the differential images have been normalized to the frame aggregation factor, frame rate and exposure settings. Thus, the intensity values in differential frames have the same proportionality to the rate of change of the light captured by the camera.

The main analysis path is to analyze the SPRM signal intensity response in small pixel areas (3x3). To estimate background changes, the differential frames are smoothed using a 2D FFT low pass filter. Thus, for each pixel area the local SPRM intensity change (signal) and the global (background) changes can be defined. In turn, the local signal can be corrected for a global background.

The authors developed the interactive software for data processing, which is based on the Python/Numpy/CUDA stack. The calculations were distributed over four Nvidia Tesla A100 GPU accelerators in a dedicated server equipped with 2x Intel Xeon Silver 4208 CPUs and 128 GB DDR4 memory. A detailed description and analysis of the data processing pipeline will be published elsewhere.

4. Literature data on dielectric permittivities of Au, Ti and Li

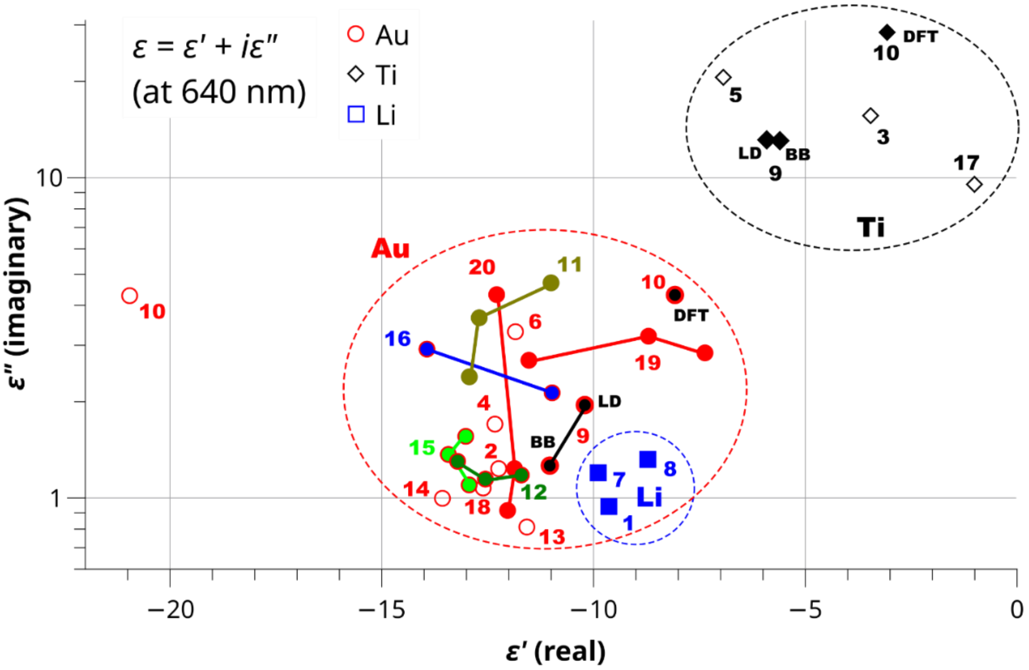

Fig. S6. Dielectric permittivities of Au, Ti and Li at 640 nm. A logarithmic scale is used for the y-axis to better visualize both Au and Ti data. Series of experimental data (gold film thickness less than 120 nm) and reported in one reference are connected by lines. BB/LD/DFT denote numerical simulation results using Brendel-Borman, Lorentz-Drude and DFT models respectively. References are ordered by date of publication.

References:

(1) Mathewson, A. G.; Myers, H. P. Absolute Values of the Optical Constants of Some Pure Metals. Phys. Scr. 1971, 4 (6), 291–292. https://doi.org/10.1088/0031-8949/4/6/009.

(2) Johnson, P. B.; Christy, R. W. Optical Constants of the Noble Metals. Phys. Rev. B 1972, 6 (12), 4370–4379. https://doi.org/10.1103/PhysRevB.6.4370.

(3) Mash, D.; Motulevich, G. P. Optical constants and electronic characteristics of Titanium. Soviet Physics JETP 1973, 36 (3), 516–520.

(4) Hagemann, H.-J.; Gudat, W.; Kunz, C. Optical Constants from the Far Infrared to the X-Ray Region: Mg, Al, Cu, Ag, Au, Bi, C, and Al_2O_3; DESY SR-74/7; DESY, 1974.

(5) Johnson, P.; Christy, R. Optical Constants of Transition Metals: Ti, V, Cr, Mn, Fe, Co, Ni, and Pd. Phys. Rev. B 1974, 9 (12), 5056–5070. https://doi.org/10.1103/PhysRevB.9.5056.

(6) Hagemann, H.-J.; Gudat, W.; Kunz, C. Optical Constants from the Far Infrared to the X-Ray Region: Mg, Al, Cu, Ag, Au, Bi, C, and Al_2O_3. J. Opt. Soc. Am. 1975, 65 (6), 742. https://doi.org/10.1364/JOSA.65.000742.

(7) Inagaki, T.; Emerson, L. C.; Arakawa, E. T.; Williams, M. W. Optical Properties of Solid Na and Li between 0.6 and 3.8 eV. Phys. Rev. B 1976, 13 (6), 2305–2313. https://doi.org/10.1103/PhysRevB.13.2305.

(8) Rasigni, M.; Rasigni, G. Optical Constants of Lithium Deposits as Determined from the Kramers-Kronig Analysis. J. Opt. Soc. Am. 1977, 67 (1), 54. https://doi.org/10.1364/JOSA.67.000054.

(9) Rakić, A. D.; Djurišić, A. B.; Elazar, J. M.; Majewski, M. L. Optical Properties of Metallic Films for Vertical-Cavity Optoelectronic Devices. Appl. Opt. 1998, 37 (22), 5271. https://doi.org/10.1364/AO.37.005271.

(10) Werner, W. S. M.; Glantschnig, K.; Ambrosch-Draxl, C. Optical Constants and Inelastic Electron-Scattering Data for 17 Elemental Metals. Journal of Physical and Chemical Reference Data 2009, 38 (4), 1013–1092. https://doi.org/10.1063/1.3243762.

(11) Gao, L.; Lemarchand, F.; Lequime, M. Comparison of Different Dispersion Models for Single Layer Optical Thin Film Index Determination. Thin Solid Films 2011, 520 (1), 501–509. https://doi.org/10.1016/j.tsf.2011.07.028.

(12) Olmon, R. L.; Slovick, B.; Johnson, T. W.; Shelton, D.; Oh, S.-H.; Boreman, G. D.; Raschke, M. B. Optical Dielectric Function of Gold. Phys. Rev. B 2012, 86 (23), 235147. https://doi.org/10.1103/PhysRevB.86.235147.

(13) Babar, S.; Weaver, J. H. Optical Constants of Cu, Ag, and Au Revisited. Appl. Opt. 2015, 54 (3), 477. https://doi.org/10.1364/AO.54.000477.

(14) McPeak, K. M.; Jayanti, S. V.; Kress, S. J. P.; Meyer, S.; Iotti, S.; Rossinelli, A.; Norris, D. J. Plasmonic Films Can Easily Be Better: Rules and Recipes. ACS Photonics 2015, 2 (3), 326–333. https://doi.org/10.1021/ph5004237.

(15) Yakubovsky, D. I.; Arsenin, A. V.; Stebunov, Y. V.; Fedyanin, D. Yu.; Volkov, V. S. Optical Constants and Structural Properties of Thin Gold Films. Opt. Express 2017, 25 (21), 25574. https://doi.org/10.1364/OE.25.025574.

(16) Ciesielski, A.; Skowronski, L.; Trzcinski, M.; Górecka, E.; Trautman, P.; Szoplik, T. Evidence of Germanium Segregation in Gold Thin Films. Surface Science 2018, 674, 73–78. https://doi.org/10.1016/j.susc.2018.03.020.

(17) Palm, K. J.; Murray, J. B.; Narayan, T. C.; Munday, J. N. Dynamic Optical Properties of Metal Hydrides. ACS Photonics 2018, 5 (11), 4677–4686. https://doi.org/10.1021/acsphotonics.8b01243.

(18) Magnozzi, M.; Ferrera, M.; Mattera, L.; Canepa, M.; Bisio, F. Plasmonics of Au Nanoparticles in a Hot Thermodynamic Bath. Nanoscale 2019, 11 (3), 1140–1146. https://doi.org/10.1039/C8NR09038F.

(19) Yakubovsky, D. I.; Stebunov, Y. V.; Kirtaev, R. V.; Ermolaev, G. A.; Mironov, M. S.; Novikov, S. M.; Arsenin, A. V.; Volkov, V. S. Ultrathin and Ultrasmooth Gold Films on Monolayer MoS 2. Adv Materials Inter 2019, 6 (13), 1900196. https://doi.org/10.1002/admi.201900196.

(20) Rosenblatt, G.; Simkhovich, B.; Bartal, G.; Orenstein, M. Nonmodal Plasmonics: Controlling the Forced Optical Response of Nanostructures. Phys. Rev. X 2020, 10 (1), 011071. https://doi.org/10.1103/PhysRevX.10.011071.

5. Influence of ion redistribution on the refractive index near the sensor surface

Given a polarizability and concentrations of the atoms/molecules/ions, that make up the media, its refractive index can be determined using the Lorentz-Lorenz relation.[iv] A very similar Clausius-Mosotti relation does the same thing for the dielectric permittivity.According to the Lorentz-Lorenz relation:

$$\frac{n^2-1}{n^2+2} = \frac{\sum N\alpha_m}{3\varepsilon_0},$$

where *n* is the refractive index of the composition, ε_0 is vacuum permittivity, *N* is the number concentration of molecules and α_m is the molecular polarizability. This equation reflects the fact that the effective refractive index of media can be split into several additive values: that of the solvent itself (ACN), and that of the anions and that of the cations.

The polarizability volumes α'_m of Li, ClO4 and ACN in 10^{-24} cm^3 units (cgs) are given as 0.03, 5.25 and 4.43 respectively.[v] The molecular polarizability α_m is proportional to the polarizability volume α'_m (in cgs units 10^{-24} cm^3):

$$\alpha_m = \frac{4\pi\varepsilon_0}{10^6}\alpha'_m$$

Therefore, the excess RI value due to the solute is determined mainly by the concentration of perchlorate anions. With a molecular weight of 41.05 g/mol and density of 0.78 g/ml, the molar concentration of the solvent (ACN) is ~19 M. Therefore, the number of molecules of the solute itself ($LiClO_4$) at concentration of 100 mM is about 190 times less than that of ACN. Therefore, their occupied volume can be neglected and the number of ACN molecules can be estimated directly from its density and molecular weight. If the RI is calculated from the mean polarizability of the molecules, the RI of pure ACN is 1.34488 and by adding 100 mM $LiClO_4$ it changes to 1.34728. So *n* increases by at most $2.4*10^{-3}$ (it should be a little lower considering the expelled volume). According to the literature, the addition of 100 mM $LiClO_4$ to C3-C5 alkanols (propanol/butanol/pentanol) resulted in an increase of their RI by ~1.5-2.2 $*10^{-3}$.[vi] The calculated RI of pure water (α'_m=1.49) is 1.33856. The RI values calculated from the molecular polarizabilities are therefore plausible.

Using this approach, the profile of refractive index can be calculated from the distribution of ions near the sensor surface. According to the Boltzmann distribution used in the Gouy-Chapman model, the concentration profile of ionic species of the 1:1 electrolyte near a charged surface depends on the electrical potential ψ and the molar bulk volume concentration *c*:

$$c_+(x) = ce^{-\frac{e_0\psi(x)}{kT}}, \quad c_-(x) = ce^{+\frac{e_0\psi(x)}{kT}};$$

where e_0 is the elementary charge, *k* is the Boltzmann constant, and *T* is the absolute temperature. According to Gouy-Chapman theory, the electric potential decays exponentially

$$\psi(x) = \psi_0 e^{-\kappa x},$$

where $\psi_0$is the surface potential and κ^{-1} is the Debye length. For 1:1 monovalent electrolyte it is given by:

$$\kappa^{-1} = \sqrt{\frac{\varepsilon\,\varepsilon_0 R\,T}{2*10^3\,F^2\,c}}$$

where *R* and *F* are the gas and Faraday constants, ε and ε_0 are the static dielectric permittivity of the media and free space respectively. It should be noted that although the refractive index and dielectric permittivity of ACN and water are quite similar in the optical range, their static dielectric permittivity is practically two times different: 37.5 and 78.4, respectively. Therefore, the Debye length in ACN is ~1.45 times shorter than in water, under otherwise the same conditions. In pure water at pH=7 and 20° C κ^{-1} is ~1 µm (due to dissociated H^+ and OH^-). As salts are dissolved, the Debye length decreases. At 1 mM concentration of 1:1 electrolyte it is only 9.5 nm, and at 100 mM it is only 0.95 nm. can be regarded as the thickness of the ion depleted / enriched layer. Within this region, the concentration of co-ions can drop from C to zero, but the concentration of counter-ions can reach a multiple of the bulk concentration. Given their different effects on the refractive index, the dependence of the SPR on the potential of the gold surface is not obvious and depends not only on the potential itself, but also on the concentration and composition of an electrolyte. Note, that the Debye length itself depends on the dielectric permittivity of the solvent. Other physicochemical processes could also be considered. [vii] [viii]

The model described above is unfortunately only applicable for linear approximation when the normalized potential [qψ/kT] <<1. Since kT corresponds to a potential of about 25 mV at room temperature, it should not be used directly to calculate the concentration profile of ions. The simple Gouy-Chapman (GC) model gives physically unreachable concentrations of ions near the charged surface, a problem that is alleviated by the addition of the Stern layer (GCS-model). On the other hand, the thickness of the depletion layer is so small with respect to the penetration depth of the evanescent field, that an exact concentration profile is not required to calculate optical effects. It is sufficient to know the total amount of excess/expelled ions within this region. This roughly corresponds to the surface charge.

6. Correction of cyclic voltarefractograms of adsorbed TiO_2 nanoparticles for background

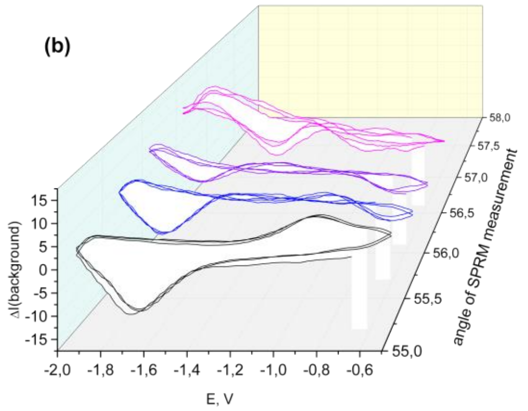

Fig. S7. Cyclic voltarefractograms of the background signal, measured at different incident angles, during potential cycling in 0.25 M $LiClO_4$ acetonitrile solution

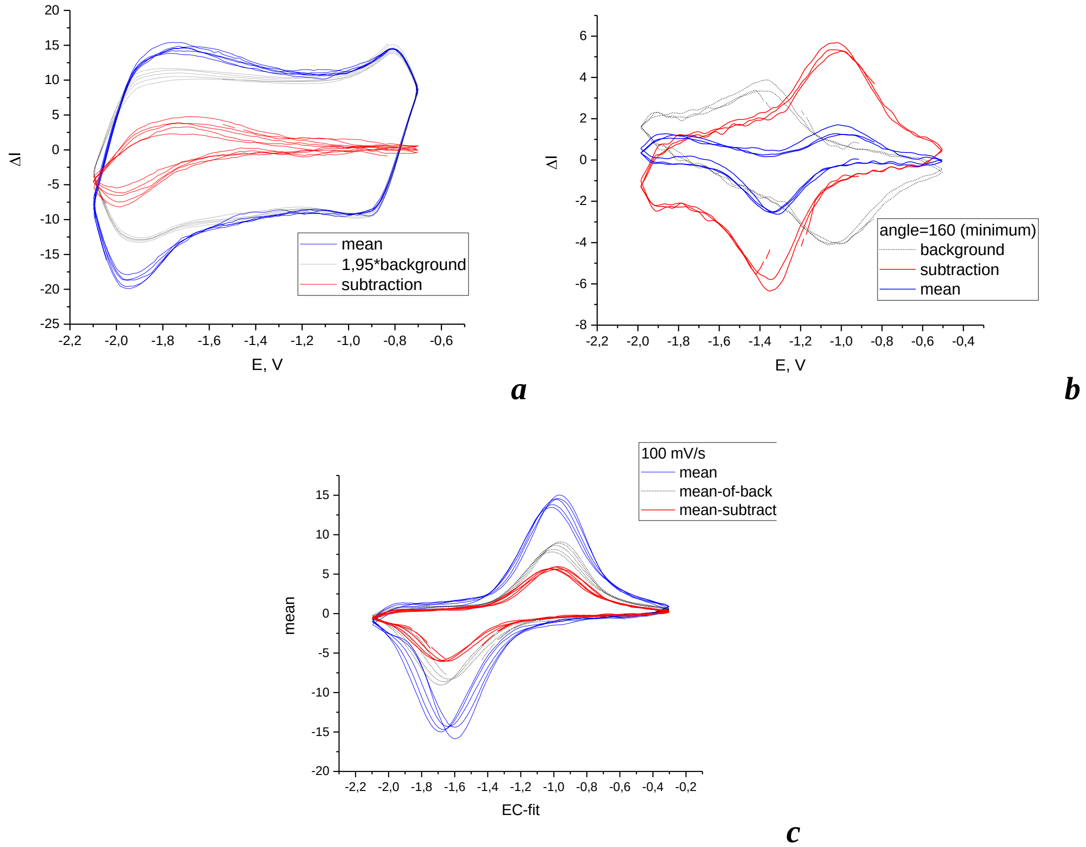

Fig. S8. Correction of cyclic voltarefractograms of adsorbed TiO_2 nanoparticles to background for a-TiO_2 + r -TiO_2 (a), a-TiO_2 + r -TiO_2 (b), anatase (c).

7. In situ cyclic voltammetry of prism coated by a-TiO_2 and r-TiO_2 NPs

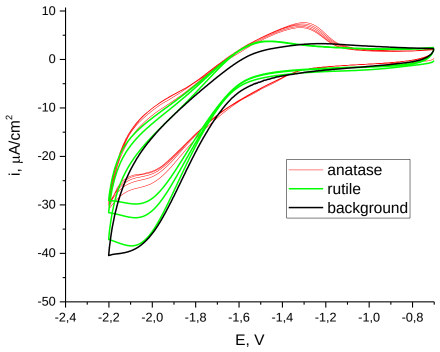

Fig. S9. In situ cyclic voltammograms of the Au-surface of prism: clean and with adsorbed r-TiO_2 and a-TiO_2 NPs in 0.25 M $LiClO_4$ acetonitrile solution at scan rate of 50 mV/s.

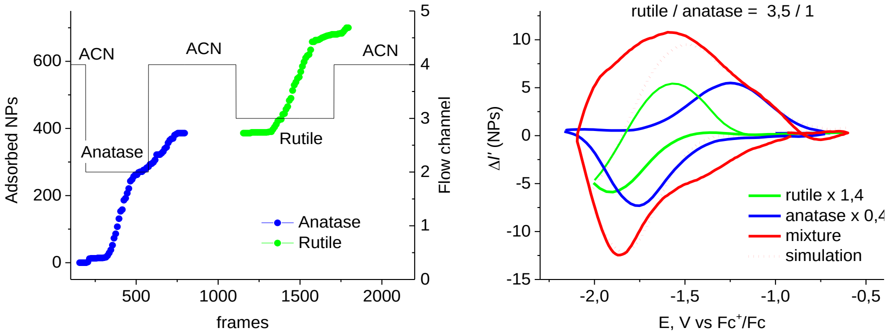

Fig. S10. WF-SPRM *in situ* counting of adsorption of a-TiO_2 and r-TiO_2 nanoparticles onto TMA-modified gold surface (a) and subsequent voltarefractograms obtained for these NPs in 0.25 M $LiClO_4$ acetonitrile solution at a scan rate of 200 mV/s (b). The simulation (red dots) of the experimental spectral signal (red line) was obtained by summing the individual spectral curves of a-TiO_2 (blue) and r-TiO_2 (green).

8. Ex situ cyclic voltammetry of a-TiO_2 and r-TiO_2 modified gold electrodes: effect of scan rate

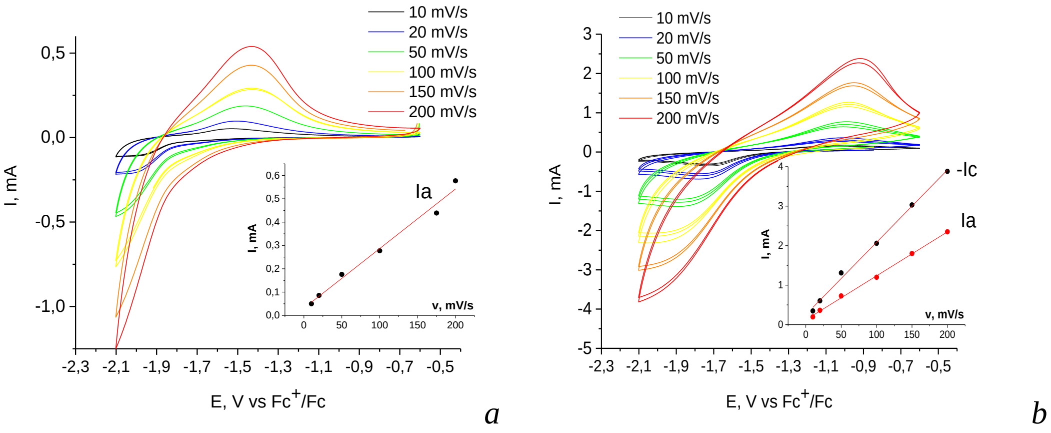

Fig. S11. Cyclic voltammograms of an Au electrode, modified with 25 µg r-TiO_2 (a) or a-TiO_2 (b) NPs in 0.25 M $LiClO_4$ acetonitrile solution at different scan rates. The dependencies of the peak currents on the scan rates are shown in the insets.

[i] Li, N.; Zhao, Y.; Wang, X.; Peng, H.; Li, G. Facile Synthesis and Enhanced Photocatalytic Properties of Truncated Bipyramid-Shaped Anatase TiO_2 Nanocrystals. *Mater. Lett.*, **2013**, *102-103*, 53-55. DOI: 10.1016/j.matlet.2013.03.106

[ii] Chen, H.-S.; Su, C.; Chen, J.-L.; Yang, T.-Y.; Hsu, N.-M.; Li, W.-R.. Preparation and Characterization of Pure Rutile TiO_2 Nanoparticles for Photocatalytic Study and Thin Films for Dye-Sensitized Solar Cells. *J. Nanomater*. **2011**, *47*, 1-8. DOI: 10.1155/2011/869618.

[iii] Ghilane, J.; Hapiot, P.; Bard, A. J. Metal/Polypyrrole Quasi-Reference Electrode for Voltammetry in Nonaqueous and Aqueous Solutions. *Anal. Chem.* **2006**, 78 (19), 6868-6872. DOI: 10.1021/ac060818o.

[iv] Kragh, H. The Lorenz-Lorentz Formula: Origin and Early History. *Substantia* **2018**, 2 (2), 7-18. doi:10.13128/substantia-56

[v] Camus, M. N.; Megnassan, E.; Proutiere, A.; Chabanel, M. An Investigation on Ionic Solvation by Refractometric Measurements and Electrostatic Interaction Calculations. *J. Mol. Struct*. **1993**, 295, 155-167. DOI: 10.1016/0022-2860(93)85017-O

[vi] Roy, M. N.; Sikdar, P. S.; De, P. Physico-Chemical Study of Lithium Perchlorate in Alkanols (C3–C5) with the Manifestation of Solvation Consequences. *J. Mol. Liq.*, **2013**, 187, 368-373. DOI: 10.1016/j.molliq.2013.09.009

[vii] Laurinavichyute, V. K.; Nizamov, S.; Mirsky, V. The Role of Anion Adsorption in the Effect of Electrode Potential on SPR Response. *Chemphyschem* **2017**, 18 (12) 1552-1560. DOI: 10.1002/cphc.201601288

[viii] Nizamov, S.; Scherbahn, V.; Mirsky, V. M. Ionic Referencing in Surface Plasmon Microscopy: Visualization of the Difference in Surface Properties of Patterned Monomolecular Layers. *Anal. Chem.* **2017**, 89 (7), 3873–78. DOI: 10.1021/acs.analchem.7b00251
